# Intracellular IL-32 regulates mitochondrial metabolism, proliferation, and differentiation of malignant plasma cells

**DOI:** 10.1016/j.isci.2021.103605

**Published:** 2021-12-13

**Authors:** Kristin Roseth Aass, Robin Mjelle, Martin H. Kastnes, Synne S. Tryggestad, Luca M. van den Brink, Ingrid Aass Roseth, Marita Westhrin, Muhammad Zahoor, Siv H. Moen, Tonje M. Vikene Nedal, Glenn Buene, Kristine Misund, Anne-Marit Sponaas, Qianli Ma, Anders Sundan, Richard WJ. Groen, Tobias S. Slørdahl, Anders Waage, Therese Standal

**Affiliations:** 1 Centre of Molecular Inflammation Research, Norwegian University of Science and Technology, Trondheim 7491, Norway; 2 Department of Clinical and Molecular Medicine, Norwegian University of Science and Technology, Trondheim 7491, Norway; 3 Department of Hematology, St.Olavs University Hospital, Trondheim 7491, Norway; 4 Department of Molecular Medicine, Institute of Basic Medical Sciences, University of Oslo, Oslo 0372, Norway; 5 Department of Hematology, Cancer Center Amsterdam, VU University Medical Center, Amsterdam 1081, the Netherlands

**Keywords:** Immunology, Cell biology, Cancer

## Abstract

Interleukin-32 (IL-32) is a nonclassical cytokine expressed in cancers, inflammatory diseases, and infections. Its expression is regulated by two different oxygen sensing systems; HIF1α and cysteamine dioxygenase (ADO), indicating that IL-32 may be involved in the response to hypoxia. We here demonstrate that endogenously expressed, intracellular IL-32 interacts with components of the mitochondrial respiratory chain and promotes oxidative phosphorylation. Knocking out IL-32 in three myeloma cell lines reduced cell survival and proliferation *in vitro* and *in vivo*. High-throughput transcriptomic and MS-metabolomic profiling of IL-32 KO cells revealed that cells depleted of IL-32 had perturbations in metabolic pathways, with accumulation of lipids, pyruvate precursors, and citrate. IL-32 was expressed in a subgroup of myeloma patients with inferior survival, and primary myeloma cells expressing IL-32 had a gene signature associated with immaturity, proliferation, and oxidative phosphorylation. In conclusion, we demonstrate a previously unrecognized role of IL-32 in the regulation of plasma cell metabolism.

## Highlights

• Intracellular IL-32 is an endogenous growth factor for malignant plasma cells
• IL-32 interacts with components of the electron transport chain
• IL-32 promotes oxidative phosphorylation
• IL-32 is expressed by immature, CD45 + highly proliferating malignant plasma cells

## Graphical abstract

**Figure:**
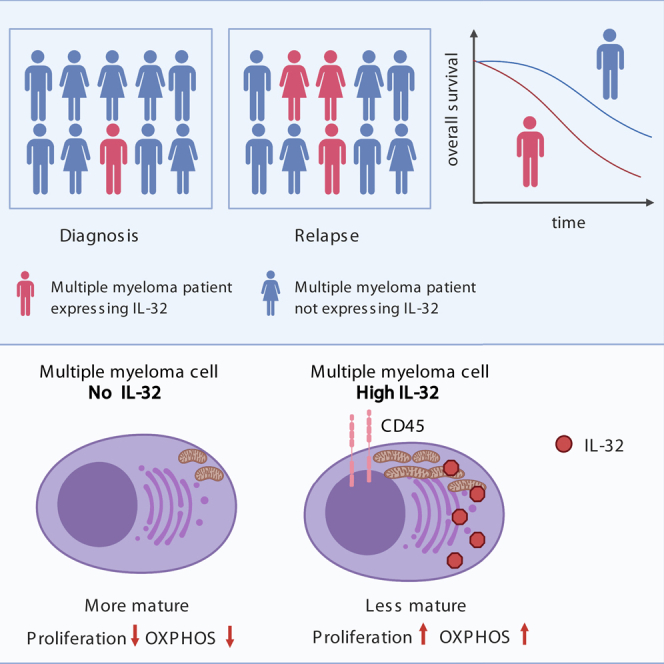

## Introduction

Multiple myeloma (MM) is a cancer of terminally differentiated plasma cells in the bone marrow. Similar to normal plasma cells, the malignant cells are dependent on the bone marrow microenvironment for survival. Most MM cell growth factors are produced by cells of stromal and hematopoietic origin, and interleukin-6 (IL-6), APRIL, and BAFF are key survival factors. Only a small number of pro-survival or proliferative factors may be produced by the cancer cells themselves (Bianchi and Munshi, 2015).

Reprogramming of cell metabolism has emerged as a central player in cancer progression, dissemination, and drug resistance. The bone marrow is characterized by areas of low oxygen levels, and the master regulator of hypoxic metabolism, HIF1α, is highly expressed in MM cells in hypoxic niches (Azab et al., 2012; Colla et al., 2010; Maiso et al., 2015). Hypoxic MM cells may exhibit a glycolytic phenotype (Ikeda et al., 2018; Maiso et al., 2015) but several studies have demonstrated that aerobic metabolism, and thus oxidative phosphorylation (OXPHOS), is fully functional in MM cells. The OXPHOS/glycolysis ratio is dynamic and possibly regulated by microenvironmental cues and state of dormancy (Birsoy et al., 2014; Marlein et al., 2019; Tevebaugh et al., 2017). Furthermore, high level of aerobic metabolism may contribute to drug resistance and disease progression in MM (Soriano et al., 2016; Zhan et al., 2017).

IL-32 is a pluripotent pro-inflammatory cytokine involved in a range of diseases including cancer, infections, and autoimmunity (Kim et al., 2005; Ribeiro-Dias et al., 2017; Aass et al., 2021). IL-32 has no sequence homology with other cytokine families, and an IL-32 receptor has not been identified. IL-32 is intriguingly regulated by two different oxygen sensing systems, HIF1α (Zahoor et al., 2017) and cysteamine (2-aminoethanethiol) dioxygenase (ADO) (Masson et al., 2019), indicating that this protein has an important function in response to low oxygen tension. We have previously shown that IL-32 is highly expressed in a subgroup of MM patients and that expression of IL-32 in MM cells is increased in response to hypoxia in a HIF1α-dependent manner (Zahoor et al., 2017). The roles and mechanisms of action of IL-32 in plasma cells is however not known.

A hallmark of multiple myeloma is the great genetic and phenotypic heterogeneity of the cancer cells. To determine the molecular function of IL-32 in malignant plasma cells, we therefore generated IL-32 KO cells from three different cell lines and characterized them by functional assays and high-throughput transcriptomic and MS-metabolomic profiling. We further identified novel binding partners to IL-32 by immunoprecipitation followed by mass spectrometry. Finally, we determined the gene expression signature of high IL-32-expressing primary MM cells from patients. We found that endogenous intracellular IL-32 promoted survival and proliferation of myeloma cells *in vitro* and *in vivo*. IL-32 interacted with components of the mitochondrial respiratory chain and acted as an important regulator of myeloma cell metabolism. Moreover, IL-32 expression in patient samples was associated with poor prognosis and an immature, proliferative plasma cell profile. Our data demonstrate a metabolic function of IL-32 and support that IL-32 is a potential prognostic biomarker and a treatment target in MM.

## Results

### IL-32 is important for myeloma cell proliferation *in vitro* and tumor engraftment *in vivo*

**Figure 1:**
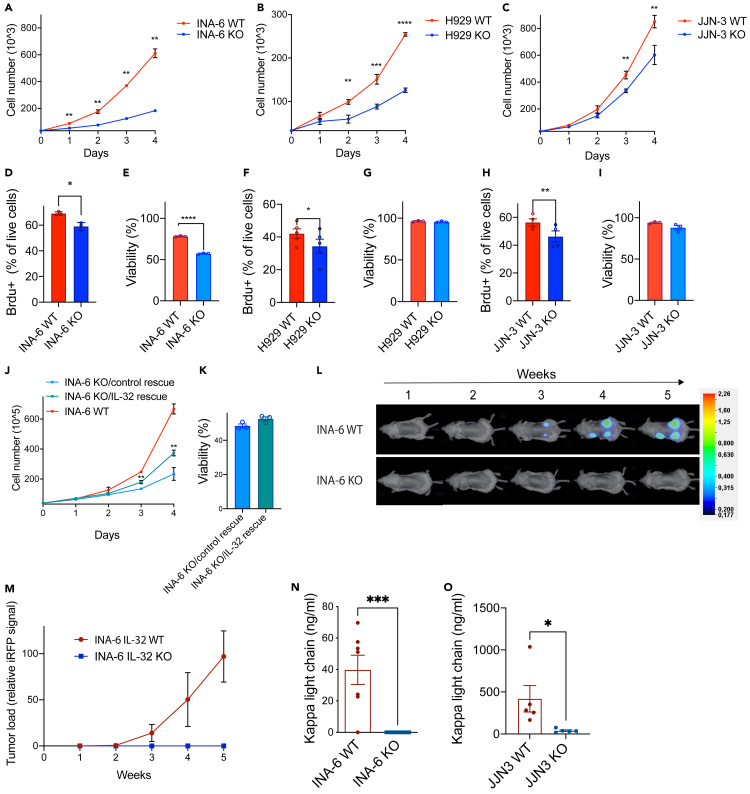
IL-32 is important for myeloma cell proliferation *in vitro* and tumor engraftment *in vivo* (A–C) INA-6, H929, and JJN-3 IL-32 KO cells were generated by CRISPR/Cas9. Proliferation of IL-32 KO and WT mock cells was assessed by automated cell counting every day for 4 days. Mean ± SD of 3 technical replicates of one representative experiment of ≥3 independent experiments are shown. Significance was evaluated by calculating mean for each day and performing multiple t tests. (D) % 5-bromo-2′-deoxyuridine(brdu)-positive live INA-6 KO and WT mock cells after 4 h. Data shown are mean ± SEM ≥3 independent experiments. Statistical significance was determined by paired Student's t test. (E) Viability of INA-6 IL-32 KO and WT mock cells was evaluated by flow cytometry using annexin/PI staining. Data shown are mean ± SEM ≥3 independent experiments. Statistical significance was determined by paired Student's t test. (F) % 5-bromo-2′-deoxyuridine(brdu)-positive live H929 KO and WT mock cells after 4 h. Data shown are mean ± SEM ≥3 independent experiments. Statistical significance was determined by paired Student's t test. (G) Viability of H929 IL-32 KO and WT mock cells was evaluated by flow cytometry using annexin/PI staining. Data shown are mean ± SEM ≥3 independent experiments. Statistical significance was determined by paired Student's t test. (H) % 5-bromo-2′-deoxyuridine(brdu)-positive live JJN-3 KO and WT mock cells after 4 h. Data shown are mean ± SEM ≥3 independent experiments. Statistical significance was determined by paired Student's t test. (I) Viability of JJN-3 IL-32 KO and WT mock cells was evaluated by flow cytometry using annexin/PI staining. Data shown are mean ± SEM ≥3 independent experiments. Statistical significance was determined by paired Student's t test. (J) IL-32 was reintroduced into INA-6 KO cells by transduction with an IL-32 lentiviral vector and proliferation of INA-6 KO/IL-32 rescue cells, and INA-6 KO/control rescue cells was assessed by cell counting. Mean ± SD of 3 technical replicates of one representative experiment of ≥3 independent experiments are shown. Significance was evaluated by calculating mean for each day and performing multiple t tests. (K) Viability of INA-6 KO/IL-32 rescue cells and INA-6 KO/control rescue was evaluated by flow cytometry using annexin/PI staining. Data shown are mean ± SEM ≥3 independent experiments. Statistical significance was determined by paired Student's t test. (L) 1 × 106 iRFP labelled INA-6 IL-32 KO and WT mock cells were implanted on humanized bone scaffolds on the flanks of RAG −/− y c−/− BALB/c mice, and tumor burden was assessed every week. The figure shows representative images of tumor burden mice injected with WT mock and KO cells. WT: N = 9, KO: N = 10. The scale bar shows the intensity of fluorescence in the 700 white channel. (M) Tumor development quantified by the pooled iRFP signal of all scaffolds. Figure shows mean ± SEM of WT: 27 scaffolds, KO: 30 scaffolds. (N) Blood was collected at the end of the experiment described in (G), and serum human kappa light chain was quantified. (O) 1 × 10^5^ JJN-3 WT (N = 5) or KO (N = 5) cells were injected into the tibia of male RAG2−/−GC−/− mice. After 20 days blood was collected, and serum human kappa light chain was quantified. ∗p ≤0.05, ∗∗p ≤0.01, ∗∗∗p ≤0.001, ∗∗∗∗p ≤0.0001.

We have previously demonstrated that IL-32 is expressed by a subgroup of MM cells (Zahoor et al., 2017). Moreover, bone marrow plasma cells obtained from healthy donors express IL-32 at higher levels relative to other B cell subsets (Figure S1A). The function of IL-32 in plasma cells is however unknown. To investigate the role of IL-32 in MM cells we depleted IL-32 using CRISPR/Cas9 from three IL-32-expressing cell lines, JJN-3, INA-6, and H929 (Figure S1B). These cell lines have different IgH translocations, t(14;16), t(11;14), and t(4;14), respectively, and also differ in terms of p53 and RAS mutations (Burger et al., 2001; Gooding et al., 1999). Strikingly, for all three cell lines, loss of IL-32 significantly reduced proliferation as assessed by automated cell counting (Figures 1A–1C) and as assessed by % live cells incorporating 5-bromo-2′-deoxyuridine (Brdu) (Figures 1D, 1F and 1H). On the other hand, depletion of IL-32 significantly reduced viability of the INA-6 KO cells compared with mock (wild-type [WT]) cells (Figure 1E) but did not affect survival of JJN-3 (Figure 1G) and H929 cells (Figure 1I). IL-6 is an important survival factor for myeloma cells (Klein et al., 1995). INA-6 is one of a few IL-6-dependent MM cell lines (Burger et al., 2001) and is also quite similar to primary myeloma cells when examined by transcriptomic correlation analysis (Sarin et al., 2020). The reduction in cell survival upon IL-32 depletion may indicate that INA-6 cells are dependent on IL-32 as a pro-survival signal in addition to IL-6.

The significant reduction in proliferation upon IL-32 depletion in all three cell lines support that IL-32 has a proliferative effect on myeloma cells. IL-32 has different isoforms (Aass et al., 2021) and based on RNA sequencing several isoforms are expressed in the three cell lines. INA-6 cells express IL-32β and IL-32γ, with the highest expression of the β-isoform (Figure S1C). To further confirm that the reduced proliferation was due to loss of IL-32 we re-introduced IL-32β in an INA-6 KO clone (INA-6 KO/IL-32 rescue) by lentiviral transduction and subsequent puromycin selection for IL-32 positive cells. INA-6 KO/IL-32 rescue cells had significantly increased proliferation compared with the INA-6 KO/control rescue cells (Figure 1J), supporting that the KO phenotype was due to lack of IL-32. Re-introduction of IL-32 did not significantly improve viability of the INA-6 KO cells (Figure 1K). Expression of IL-32 in the knock-in cells was confirmed by qPCR and western blotting (Figures S1D and E). Treating the cells with rhIL-32β and γ had no effect on survival or proliferation of INA-6 cells (Figures S1F and G), nor did it induce proliferation of JJN-3 cells (Figure S1H). rhIL-32 was biological active because it induced TNFα production in macrophages (Figure S1I). Thus, intracellular IL-32, rather than exogenous IL-32, is responsible for the proliferative effect of IL-32 in plasma cells.

Myeloma cell growth and survival are aided by factors secreted from cells in the BM microenvironment. To address if the loss of IL-32 affected the cells' abilities to establish tumors *in vivo* we performed two experiments. We first explored if the reduction in proliferation and survival of IL-32 in INA-6 KO cells *in vitro* could be compensated by factors produced by a human bone-marrow-like environment. Thus, we implanted 1 × 10^6^ INA-6 iRFP-labelled IL-32 KO and WT cells into humanized bone scaffolds in immune compromised female RAG2^−/−^ GC^−/−^mice and followed tumor growth by imaging (Groen et al., 2012; Westhrin et al., 2020). Cell injections were successful for all mice because fluorescence was detected in all scaffolds at day 0, but only cells expressing IL-32 engrafted (Figures 1L and 1M). Immunoglobulin kappa light chain is secreted from the tumor cells, and levels of kappa light chain in serum are commonly used as a tumor marker. In line with the imaging data, kappa light chain was undetectable in mice implanted with INA-6 KO cells (Figure 1N). We next explored if depletion of IL-32 from the more aggressive and robust cell line JJN-3 affected tumor growth *in vivo*. In contrast to INA-6 cells, which are dependent on a human bone marrow microenvironment, JJN-3 cells do engraft in murine bone marrow (Hjorth-Hansen et al., 1999). Thus, we injected 1 × 10^5^ JJN-3 IL-32 WT or KO cells into the tibiae of male RAG2^−/−^GC^−/−^ mice. After 20 days blood was collected, and serum human kappa light chain was quantified. Human kappa light chain was detected in all mice, but it was significantly reduced in mice injected with IL-32 KO cells (Figure 1O). Hence, loss of IL-32 in the MM cells cannot be compensated by microenvironmental-derived factors, and myeloma cells lacking IL-32 have reduced tumorigenic potential *in vivo*.

### IL-32 is localized to the mitochondria and interacts with components of the mitochondrial respiratory chain

**Figure 2:**
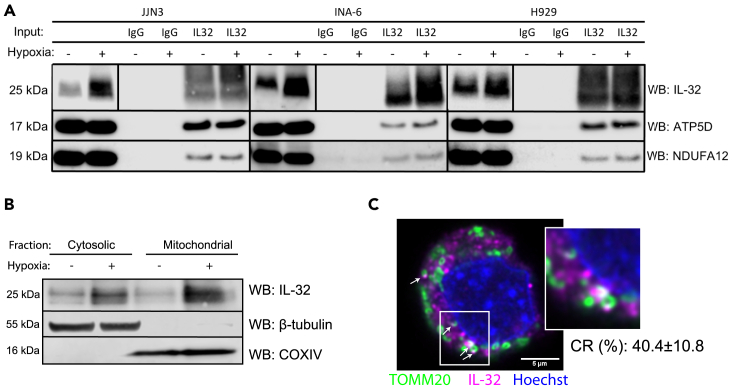
IL-32 is localized to the mitochondria and interacts with components of the mitochondrial respiratory chain (A) CO-IP was performed by pull-down of endogenous IL-32 in INA-6, JJN-3, and H929 cells. Representative immunoblots of ATP5D, NDUFA12, and IL-32 are shown. The vertical lines in the IL-32 lanes are to indicate that to improve visualization contrast/brightness were adjusted differently for the total cell lysate (2 lanes to the left) and for the IP samples (4 lanes to the right). (B) Representative immunoblot of IL-32 in the mitochondrial and cytosolic fraction of JJN-3 cells cultured in normoxia (20% oxygen) and hypoxia (2% oxygen). (C) Representative confocal image of hypoxic JJN-3 cells stained for IL-32 (magenta, Alexa 647), mitochondria (TOMM20, green, Alexa 488), and nucleus (blue, Hoechst). Imaging was performed with a Leica SP9, using a 63 × 1.4 (oil) objective and LAS X software and deconvoluted using Huygens. Scale bar: 5μM. Arrows indicate areas of colocalization of TOMM20 and IL-32. Correlation rate (CR, in %) is the mean ± SD calculated from N = 4 images analyzed in Leica Application Suite X.

An IL-32 receptor is not identified, and it is not entirely clear how IL-32 acts at the molecular level (Aass et al., 2021). Thus, to identify IL-32 binding partners we performed co-immunoprecipitation of endogenously expressed IL-32 followed by mass spectrometry analyses of the precipitates. Pull-down was performed on lysates from cells cultured for 24 h in hypoxic conditions (2% O_2_) to increase IL-32 protein expression (Zahoor et al., 2017). IL-32 KO cells were used as pull-down control to increase the specificity of the analysis. Intriguingly, 7 of 33 proteins identified to bind to IL-32 were mitochondrial proteins (Table 1). Considering the proportion of mitochondrial proteins in the human proteome, this is more than could be expected by chance (chi square test with yate's correction p = 0.0005). The interacting proteins included a subunit of the ATP synthase (ATP5D), a subunit of the NADH:ubiquinone oxidoreductase (NDUFA12), which is part of the respiratory complex (RC) I subunit, and a subunit of RC III, ubiquinol-cytochrome c reductase (UQCR11). IL-32 also interacted with dihydroorotate dehydrogenase (DHODH), which associates with RC III in the inner mitochondrial membrane (Fang et al., 2013). IL-32 also pulled down the mitochondrial transporters (ABCB6 and ABCB10), involved in heme synthesis and oxidative stress response (Bayeva et al., 2013; Krishnamurthy et al., 2006). Interactions of IL-32 with ATP5D and NDUFA12 were verified by IP western blotting for the INA-6, H929, and JJN-3 cells (Figure 2A), supporting results from the IP-MS analysis. Due to lack of suitable antibodies reverse IP with NDUFA12 and ATP5D was not possible. We were, however, able to pull down IL-32 using an antibody toward the ATP synthase complex (Figure S2), further supporting an association between IL-32 and the ATP synthase. Localization of IL-32 to the mitochondria was confirmed by the presence of IL-32 in the mitochondrial fraction of cell lysates (Figure 2B). IL-32 was also found colocalized with mitochondria at distinct sites by confocal microscopy (colocalization rate for JJN3: 40.43% ± SD 10.87, INA-6: 39.98 ± SD 20.63, and H929: 39.13 ± SD 5.39) (Figure 2C).

**Table 1:** Proteins identified as interaction partners for IL-32

ProteinAcc	Entrez ID	Organism	Full name and gene symbol^a^
O00308	11060	Homo sapiens	WW domain containing E3 ubiquitin protein ligase 2 (WWP2)
**O14957**	10975	**Homo sapiens**	**Ubiquinol-cytochrome c reductase, complex III subunit XI (UQCR11)**
O43752	10228	Homo sapiens	Syntaxin 6 (STX6)
O75844	10269	Homo sapiens	Zinc metallopeptidase STE24 (ZMPSTE24)
O76094	6731	Homo sapiens	Signal recognition particle 72 (SRP72)
P10321	3107	Homo sapiens	Major histocompatibility complex, class I C (HLA-C)
P20645	4074	Homo sapiens	Mannose-6-phosphate receptor, cation dependent (M6PR)
P24001	9235	Homo sapiens	Interleukin 32 (IL32)
**P30049**	513	**Homo sapiens**	**ATP synthase, H+ transporting, mitochondrial F1 complex, delta subunit (ATP5D)**
P30460	3106	Homo sapiens	Major histocompatibility complex, class I, B (HLA-B)
P33908	4121	Homo sapiens	Mannosidase alpha class 1A member 1 (MAN1A1)
P51795	1184	Homo sapiens	Chloride voltage-gated channel 5 (CLCN5)
**Q02127**	1723	**Homo sapiens**	**Dihydroorotate dehydrogenase (quinone) (DHODH)**
Q08188	7053	Homo sapiens	Transglutaminase 3 (TGM3)
Q09470	3736	Homo sapiens	Potassium voltage-gated channel subfamily A member 1 (KCNA1)
Q15904	537	Homo sapiens	ATPase H+ transporting accessory protein 1 (ATP6AP1)
**Q5VTU8**	432369	**Homo sapiens**	**ATP synthase, H+ transporting, mitochondrial F1 complex, epsilon subunit pseudogene 2 (ATP5EP2)**
Q68DH5	92255	Homo sapiens	LMBR1 domain containing 2 (LMBRD2)
Q9BW60	64834	Homo sapiens	ELOVL fatty acid elongase 1 (ELOVL1)
Q9BXS4	9528	Homo sapiens	Transmembrane protein 59 (TMEM59)
**Q9NP58**	10058	**Homo sapiens**	**ATP binding cassette subfamily B member 6 (Langereis blood group) (ABCB6)**
Q9NPD3	54512	Homo sapiens	Exosome component 4 (EXOSC4)
**Q9NRK6**	23456	**Homo sapiens**	**ATP binding cassette subfamily B member 10 (ABCB10)**
**Q9UI09**	55967	**Homo sapiens**	**NADH:ubiquinone oxidoreductase subunit A12 (NDUFA12)**
Q9Y2Q5	28956	Homo sapiens	Late endosomal/lysosomal adaptor, MAPK and MTOR activator 2 (LAMTOR2)
Q9Y5U9	51124	Homo sapiens	Immediate early response 3 interacting protein 1 (IER3IP1)
O43861-1	374868	Homo sapiens	Probable phospholipid-transporting ATPase IIB
P25788-1	5684	Homo sapiens	Proteasome subunit alpha type-3
Q08554-1	1823	Homo sapiens	Desmocollin-1
Q86VZ5-1	259230	Homo sapiens	Phosphatidylcholine:ceramide cholinephosphotransferase 1
Q93050-1	57130	Homo sapiens	V-type proton ATPase 116 kDa subunit a isoform 1
Q9BXP2-1	56996	Homo sapiens	Solute carrier family 12 member 9
Q9ULH0-1	57498	Homo sapiens	Kinase D-interacting substrate of 220 kDa

a Proteins detected in MS-analysis of pull-down of endogenous IL-32 from hypoxic JJN-3 cells. Interaction partners were identified by excluding all MS target proteins that were not detected in all of 5 IL-32 pull-down replicates and corresponding pull-downs of IL-32 in IL-32 KO cells. Mitochondrial proteins highlighted in bold.

### IL-32 enhances mitochondrial respiration

**Figure 3:**
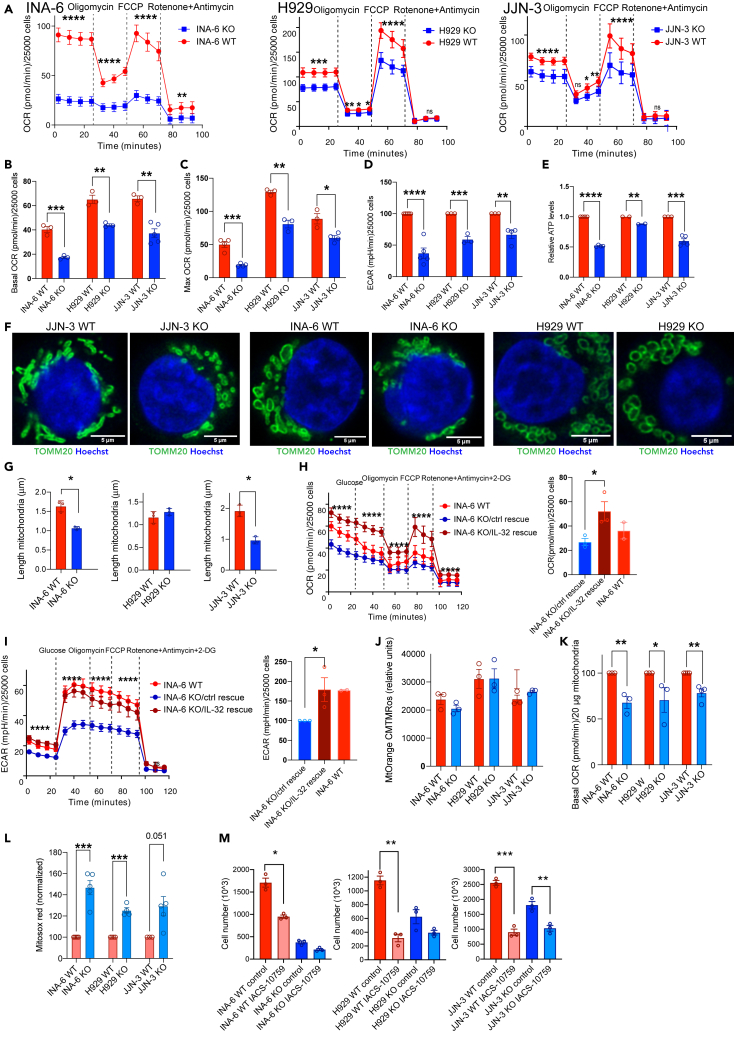
IL-32 enhances mitochondrial respiration (A) Representative Seahorse Mito Stress Test assay measuring OCR in INA-6, H929, and JJN-3 KO and WT mock. Four first measurements show basal OXPHOS, after injection of oligomycin: non-ATP oxygen consumption (proton leak), after FCCP injection: maximal OCR, after injection of rotenone and antimycin: nonmitochondrial respiration. Data show mean ± SD of 20 technical replicates. The differences between KO and WT mock cells were significant using two-way ANOVA and Sidàk's multiple comparison test (p ≤0.0001). (B) Mean basal respiration (basal OCR) in INA-6, H929, and JJN-3 KO and WT mock cell lines. Data shown are mean ± SEM of 3 independent experiments. (C) Mean maximal respiration (max OCR) in INA-6, H929, and JJN-3 KO and WT mock cell lines. Data shown are mean ± SEM of 3 independent experiments. (D) Mean basal glycolysis (±SEM) in IL-32 KO and WT cell lines analyzed by Seahorse Glycolysis Stress Test measuring ECAR. Data shown are mean ± SEM of 3 independent experiments. (E) Relative ATP levels in INA-6, H929, and JJN-3 KO and WT mock cells quantified by CTG-assay. Data shown are mean ± SEM of 3 independent experiments. (F) Representative confocal images of mitochondria of IL-32 JJN-3 KO and WT mock cells stained for TOMM20 (green, Alexa 488) and nuclei (Hoechst, blue). Imaging was performed with a Leica SP9, using a 63 × 1.4 (oil) objective and LAS X software and deconvoluted using Huygens. Scale bar: 5μM. Arrows indicate areas of colocalization of TOMM20 and IL-32. (G) Length of mitochondria in INA-6, H929, and JJN-3 IL-32 KO and WT mock cells analyzed in Fiji Software. Data are presented as mean length (u m) ±SEM of mitochondria imaged with the same staining as in (F) in 3 independent experiments (see STAR Methods for details). (H) Representative graph showing OXPHOS in INA-6 KO/IL-32 rescue cells and IL-6 KO/rescue control (mean ± SD of more than 20 technical replicates). The difference between INA-6 control rescue and INA-6 IL-32 rescue was significant using two-way ANOVA (P ≤0.0001). Bar plot shows mean basal OCR (±SEM) of 3 independent experiment. INA-6 WT mock cells were included for comparison. (I) Representative graph showing glycolysis in INA-6 KO/IL-32 rescue cells and INA-6 KO/control rescue cells (mean ± SD) of more than 20 technical replicates. The difference between INA-6 KO/control rescue cells and INA-6 KO/IL-32 rescue cells was significant using two-way ANOVA and Sidàk's multiple comparison test (P ≤0.0001). The bar plot shows mean basal glycolysis (ECAR) (±SEM) of 3 independent experiment. INA-6 WT mock cells were included for comparison. (J) Membrane potential in isolated mitochondria from IL-32 KO and WT mock cells quantified by Mitotracker Orange CMTMRos fluorescence. The bar plots show mean ± SEM of 3 independent experiments. (K) Mean basal respiration (basal OCR) in isolated mitochondria from INA-6, H929, and JJN-3 KO and WT mock cell lines. Data are shown as mean ± SEM of 3 independent experiments. (L) Mitochondrial ROS in INA-6, H929, and JJN-3 KO and WT mock cell lines quantified by Mitosox Red staining. Figure shows Mitosox fluorescence of KO and WT cells normalized to WT for each independent experiment (N >3). Data are shown as mean ± SEM. (M) INA-6, H929, and JJN-3 IL-32 KO and WT mock cells were grown in medium supplemented with IACS-10759 (10 nM), and number of cells was determined by automated counting after 4 days of culture. Data shown are mean total number of cells ±SEM of 3 independent experiments. Difference in proliferation between untreated control and inhibitor-treated samples was assessed for KO and WT mock cells by RM one-way ANOVA followed by Sidak's multiple comparison test. ns, not significant; ∗p ≤0.05, ∗∗p ≤0.01, ∗∗∗p ≤0.001, ∗∗∗∗p ≤0.0001.

To investigate if IL-32 regulates mitochondrial respiration we measured oxidative phosphorylation (OXPHOS) by quantifying the oxygen consumption rate (OCR). OCR was significantly reduced in all three IL-32 KO cell lines (Figure 3A). The IL-32-expressing cells respired significantly more than KO cells both in basal culture conditions (Figure 3B) and when maximum respiration was triggered by FCCP (Figure 3C), supporting that IL-32 promotes OXPHOS in MM cells. Glycolysis, as measured by extracellular acidification rate (ECAR), was also significantly reduced in KO cells compared with WT cells (Figure 3D). Thus, aerobic glycolysis did not seem to be increased to compensate for the lack of aerobic respiration. In line with the reduction in OCR, intracellular ATP was reduced in IL-32-KO cells compared with WT cells (Figure 3E). The mitochondria in JJN-3 and INA-6 KO cells appeared rounded and small, compared with the more elongated, fused mitochondria of WT cells (Figure 3F). Indeed, individual mitochondria in JJN-3 and INA-6 WT cells were significantly longer than mitochondria in the KO cells (Figure 3G). However, neither the amount mitochondria (Figure S3A) nor the mitochondria membrane potential was changed (Figure S3B). Thus, the reduction in OCR and ATP production was due to less efficient OXPHOS in the mitochondria rather than a general depolarization of mitochondria or reduced amount mitochondria in the KO cells. Transfection of IL-32β into INA-6 KO cells led to expression of IL-32β in both cytosol and mitochondria (Figure S3C). The INA-6 KO/IL-32 rescued cells had improved metabolic capacity as both OCR and ECAR were increased (Figures 3H and 3I), supporting that the metabolic phenotype was due to lack of IL-32.

To further explore the effect on IL-32-depletion on mitochondrial function we isolated mitochondria from WT and KO cells. In line with the results performed on whole cells (Figure S3B) the membrane potential did not differ in mitochondria isolated from WT and KO cells in the three cell lines (Figure 3J). Importantly, however, isolated mitochondria from KO cells showed reduced OCR (Figure 2K). These findings support that the reduced OCR was related to a reduced efficiency of mitochondrial respiration and not a result of reduced availability of TCA substrate (pyruvate) from glycolysis or other anaplerotic substrates. Despite reduced OCR (Figure 3B), a significant increase in mitochondrial ROS (mtROS) was measured in whole cells (Figure 3L). There were also more ROS in isolated mitochondria (Figure S3D). Increased ROS may be due to electron leak in the mitochondrial electron transport chain when electrons exit prior to the reduction of oxygen to water at cytochrome c oxidase (Guo et al., 2016). Increased mtROS, despite of reduced OCR/ATP synthesis, may thus be due to dysfunctional/suboptimal ETC.

To assess the importance of OXPHOS for myeloma cell proliferation, we treated KO and WT cells with the OXPHOS inhibitor IACS-10759 (Molina et al., 2018). Inhibiting OXPHOS significantly reduced proliferation of all cell lines, supporting that efficient OXPHOS is needed for maximum myeloma cell proliferation. It also appeared that the KO cells were slightly less affected by the OXPHOS inhibitor, supporting that IL-32-expressing cells have higher OXPHOS activity (Figure 3M).

### Loss of IL-32 leads to perturbations in metabolic pathways

**Figure 4:**
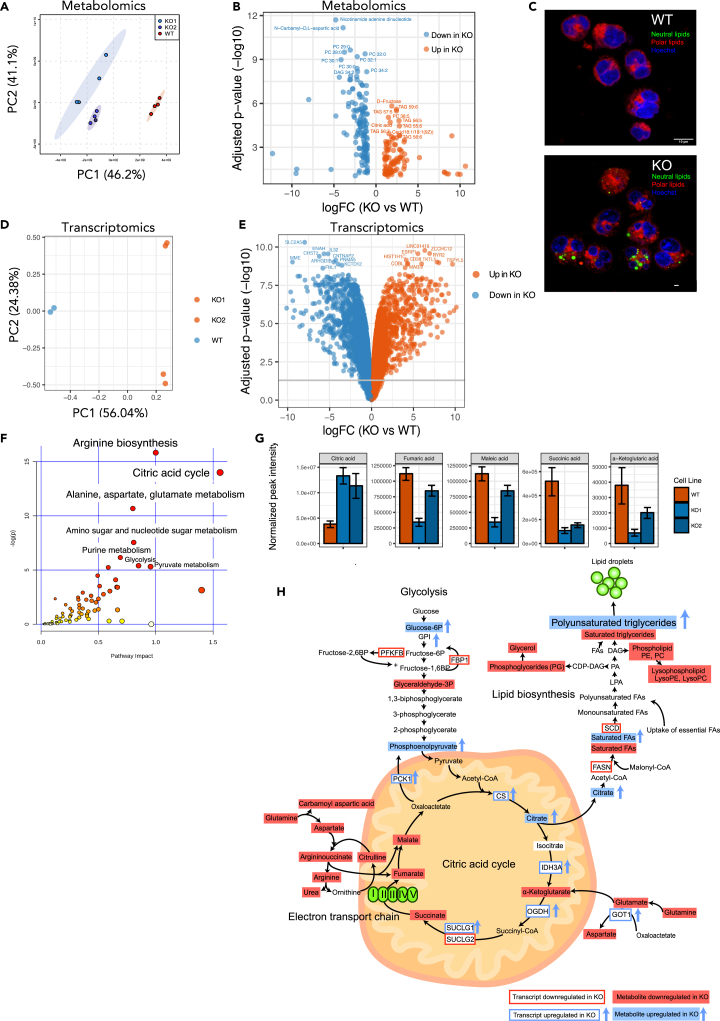
Loss of IL-32 leads to perturbations in metabolic pathways (A) PCA plot of metabolomes from two clones of INA-6 KO cells and WT mock cells. (B) Volcano plot showing significant different metabolites (p <0.05) between KO cells and WT mock cells (metabolite expression from replicates from two KO clones were merged) See also Table S1. Significance was determined by two-sided Student's t test using MetaboAnalyst 4.0 software. (C) Representative image of lipid droplets in INA-6 IL-32 KO and WT mock cells, stained with Nile Red and Hoechst. Polar lipids (red) were excited at 590 nm (600–700 nm) and neutral lipids (green) at 488 nm (500–580 nm). Confocal imaging was performed with a Leica TCS SP8 STED 3X, using a 63 × 1.4 (oil) objective and LAS X software. Scale bar: 10 μM. See Figure S4 for overview images. (D) Two INA-6 KO cell lines and WT mock cells were subjected to RNA sequencing, and the PCA plot shows the overall differences in gene expression between KO cells and WT mock cells. (E) Volcano plot showing the most significantly upregulated and downregulated genes in INA-6 KO cells (2 clones) versus WT mock cells. Statistical significance analyzed by Linear Models for Microarray Analysis (limma) in R with Benjamini-Hochberg-adjusted p values. See also Table S2 for complete gene list. (F) Joint pathway analysis (SMPDB pathways, MetaboAnalyst 4.0) of transcriptomic and metabolomic data from 2 INA-6 IL-32 KO clones and WT mock cells. The inverse logged p-value of the different pathways is shown on the y-axis, and the size and color on the dots (increased size and increasingly red) correspond to the increased inverse log p-value. Significance was determined by two-sided Student's t test using MetaboAnalyst 4.0 software. The joint pathway analysis is based on metabolites in Table S1 and genes (fold change >0.5 or < −0.5 and adjusted p value <0.05) in Table S2. (G) Significantly (p <0.05) altered citric acid cycle intermediates in two KO clones (KO1, KO2) versus WT mock cells (See also Table S1). Data are presented as mean peak intensity ± SD of 4 replicates. (H) Illustration of significantly differentially expressed genes and metabolites from the most enriched pathways in the joint pathway enrichment analysis in (F) Significance was determined by two-sided Student's t test using MetaboAnalyst 4.0 software.

To further characterize how IL-32 influences mitochondrial function and cancer cell metabolism, we characterized the metabolome and transcriptome of INA-6 WT and KO cells from two different clones using mass spectrometry and next-generation RNA-sequencing, respectively. The INA-6 cell line was chosen because it is IL-6 dependent and relatively similar to primary cells (Sarin et al., 2020). We observed major differences in metabolites between KO and WT cells (Figure 4A). There was a striking accumulation of polyunsaturated triglycerides (TAGs) in the KO cells; 36 of the 89 significant upregulated metabolites were TAGs (Figure 4B and Table S1). D-fructose and citrate were also on top of the list of metabolites increased in KO cells (Figure 4B). On the other hand, 85 of 220 downregulated metabolites in KO cells were membrane lipids (Table S1), including phosphatidylethanolamines (PE), phosphatidylcholines (PC), diacylglycerols (DAG), and saturated TAGs (Figure S4A). This indicates that fatty acid synthesis is skewed in KO cells and that fatty acids are used for synthesis of unsaturated triglycerides rather than membrane lipids. Indeed, when staining for neutral lipids in JJN-3 and INA-6 KO cells, we observed a striking accumulation of lipid droplets not present in the WT cells (Figures 4C and S4B). According to the Metabolite Set Enrichment Analysis (MSEA), the metabolic pathways aspartate metabolism, urea cycle, purine metabolism, the citric acid cycle, PC biosynthesis, the mitochondrial electron transport chain, and Warburg effect were downregulated in KO cells (Figure S4C).

RNA sequencing of the INA-6 cell line also demonstrated marked differences in gene expression between IL-32 WT and KO cells (Figures 4D and 4E, Table S2). Downregulated genes resulting from loss of IL-32 included genes involved in biological processes such as “cotranslational protein targeting to the membrane,” “nuclear-transcribed mRNA catabolic process, nonsense-mediated decay,” and “translational initiation.” In addition, terms related to immune cell proliferation, immune activation, and leukocyte differentiation were higher expressed in the WT cells (Figure S5A). In contrast, biological pathways that were upregulated in INA-6 KO cells included “translational termination,” mitochondrion organization,” and “regulation of G2/M transition of mitotic cell cycle.” Overall, genes related to processes in the mitochondria, cell division, and protein synthesis/turnover were prominently upregulated in KO cells (Figure S5B). Considering the phenotype of the KO cells, it is likely that some of the genes involved in these biological processes are upregulated in the KO cells as a compensatory mechanism.

By combining the metabolomics and transcriptomics data for INA-6 cells in a joint pathway analysis, we found arginine biosynthesis, citric acid cycle and alanine, aspartate, and glutamate metabolism to differ the most between WT and KO cells (Figure 4F). For the individual metabolites, citrate was the only upregulated intermediate in the citric acid cycle in the KO cells, whereas α-ketoglutarate, succinate, fumarate, and malate were all downregulated (Figure 4G and Table S1), indicating that the citric acid cycle is disrupted at this point in IL-32 KO cells. Limited oxidation in the electron transport chain may lead to enhanced transport of citrate out from the mitochondria and used for synthesis of fatty acids (Martínez-Reyes and Chandel, 2020) (Figure 4H). Supporting our experimental data, ATP was reduced in the KO cells (Table S1), and NAD was the most significantly downregulated metabolite in the KO cells (Figure 4B), indicative of less active mitochondrial metabolism in the KO cells. Taken together, changes in citric acid cycle intermediates, arginine biosynthesis, and fatty acid accumulation indicate dysfunctional mitochondrial OXPHOS in IL-32 KO cells.

### IL-32 expression in primary MM cells is associated with inferior survival, cell division, and oxidative phosphorylation

**Figure 5:**
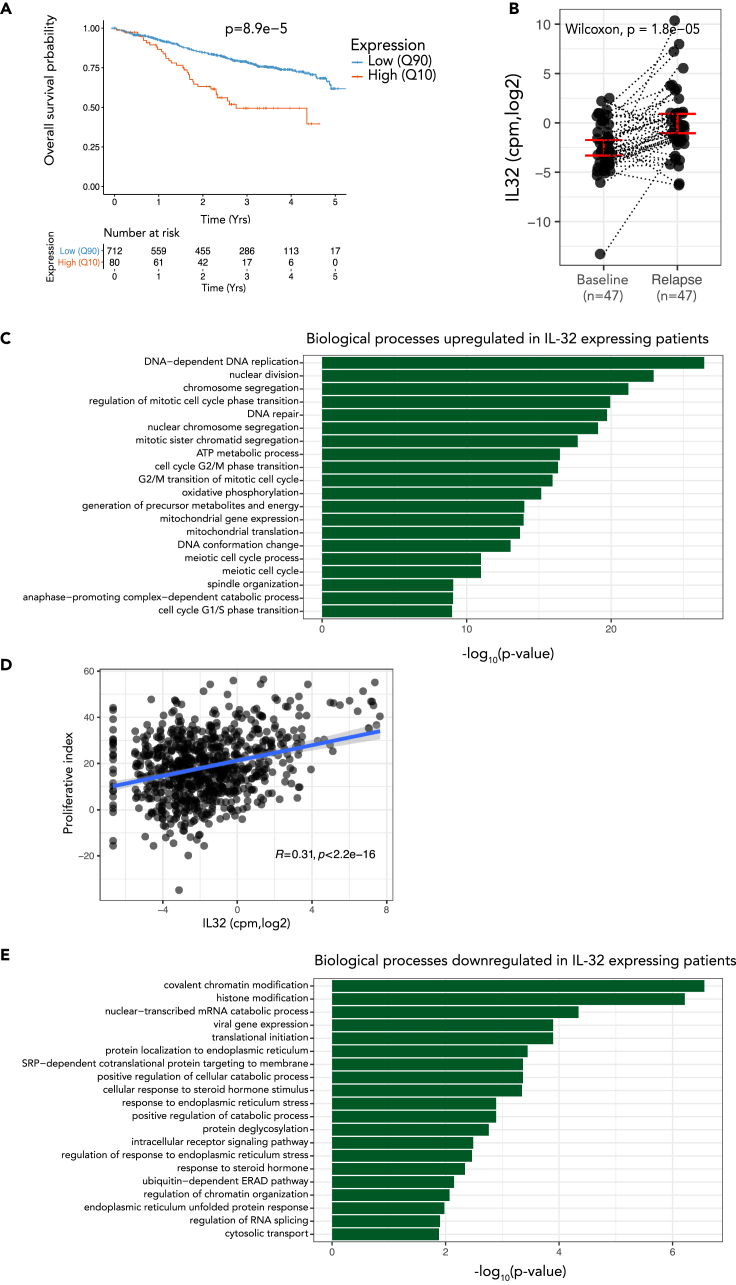
IL-32 expression in primary myeloma cells is associated with inferior survival, cell division, and oxidative phosphorylation (A) Overall survival of IL-32 expressing patients (10th percentile) compared with nonexpressing patients (90th percentile) in the IA13 CoMMpass dataset P = 8.9e-5, using Cox proportional-hazards regression model. (B) IL-32 expression in individual patients at diagnosis and first relapse in RNA-sequenced CD138^+^ cells from CoMMpass IA13. Significance was determined by Wilcoxon signed-rank test. (C) GO-analysis of the upregulated genes (Benjamini-Hochberg-adjusted p value <0.05; log2 fold change >0 for up-regulated genes) in IL-32-expressing patients (10th percentile) compared with IL-32 nonexpressing patients (90th percentile). Top significantly enriched biological processes upregulated in IL-32 expressing patients are shown. The GO terms are ordered by the Benjamini-hochberg adjusted p values. See also Tables S3 and S4. (D) Correlation between *IL32* and a proliferative index gene signature (calculated as the sum of expression values of the gene set as described in Hose et al. (2009). (E) GO-analysis of the downregulated genes (Benjamini-Hochberg-adjusted p value <0.05; log2 fold change <0 for down-regulated genes, respectively) in IL-32-expressing patients (10th percentile) compared with IL-32 nonexpressing patients (90th percentile). Top significantly enriched biological processes downregulated in IL-32 expressing patients are shown. The GO terms are ordered by the Benjamini-Hochberg adjusted p values. See also Tables S3 and S4.

We have previously shown that a subgroup of 10% to 15% of MM patients express IL-32 and that high IL-32 expression in patients associates with reduced progression-free survival (Zahoor et al., 2017) (Figures S6A and S6B). To further validate IL-32 as a prognostic factor, we analyzed overall survival of IL-32-expressing patients (upper 10th percentile, N = 80) and IL-32 nonexpressors (lower 90th percentile, N = 712) in the MMRF-CoMMpass IA13 dataset. Indeed, IL-32 expressors live significantly shorter (1005 days median survival) compared with nonexpressors (median survival not reached, P = 8.9e-5) (Figure 5A). IL-32 expression also retained prognostic information when adjusting for ISS stage (Figure S6C). Moreover, when analyzing paired diagnosis and progression samples from the same dataset, IL-32 was significantly increased upon relapse in individual patients (Figure 5B).

We next examined the characteristics of IL-32-expressing primary MM cells in terms of gene expression. In the MMRF-CoMMPass IA13 dataset, there were 4,548 significantly differentially expressed genes between IL-32-expressing (upper 10th percentile) and nonexpressing patients (lower 90th percentile) (Table S3). Interestingly, the GO enrichment analysis of differentially expressed genes revealed changes in similar GO biological processes as associated with expression of IL-32 in the cell lines: the most **upregulated** genes in IL-32-expressing patients were associated with cell division (Figure 5C and Table S4), indicating that this is indeed a signature of IL-32, both in cell lines and in primary cells. Moreover, IL-32 expression correlated with expression of genes associated with a high proliferative index in myeloma (Hose et al., 2009) (Figure 5D). ATP metabolic processes and oxidative phosphorylation were also significantly enriched in IL-32-expressing cells, supporting that IL-32-expresssing cells have active OXPHOS as compared with nonexpressing cells. In line with previous published data (Zahoor et al., 2017), we found IL-32 expression to be highly correlated with HIF1α expression (Figures S6D and S6E). Genes **downregulated** in IL-32-expressing patients were associated with protein handling and endoplasmic reticulum stress, biological processes related to the high immunoglobulin secretion from terminally differentiated plasma cells (Figure 5E).

**Figure 6:**
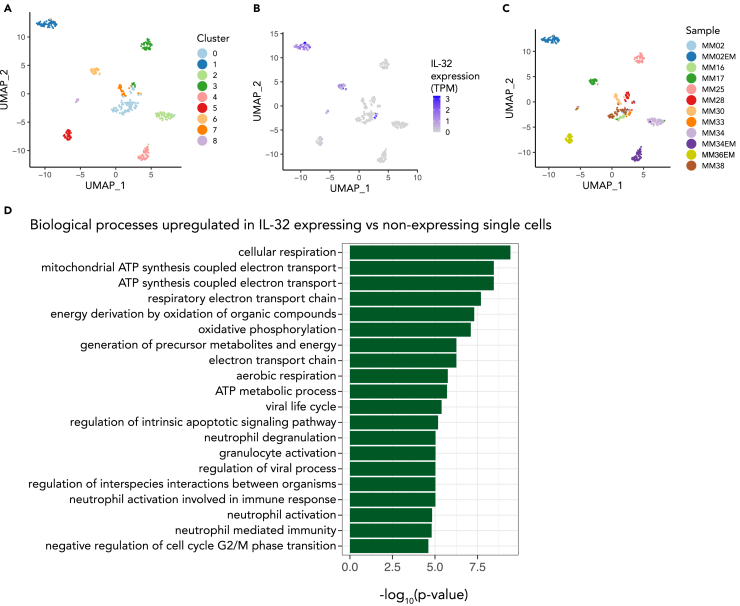
Single cell transcriptome analysis of IL-32-expressing myeloma cells (A) Uniform manifold approximation and projection (UMAP) plot colored by the identified cell clusters from a single-cell dataset (GSE106218) with primary myeloma cells. Analyzed with Seurat package in R. (B) UMAP plot colored by the level of IL32-expression per cell. (C) UMAP plot colored by patient sample. (D) Top 20 gene ontology terms (biological processes) for genes enriched in IL-32 expressing patient cells. The GO terms are ordered by the Benjamini-Hochberg adjusted p values. The data were obtained from Ryu et al. (Ryu et al., 2020).

To investigate the distribution of IL-32 gene expression within the malignant plasma cell population and to see if the highly proliferating, respiratory phenotype is directly linked to IL-32 expression within the same cell, we analyzed a publicly available single-cell dataset of MM cells sampled from bone marrow and extramedullary tumors (Ryu et al., 2020). We identified nine distinct clusters across the 12 patient samples with a total of 488 single cells of which IL-32 was mainly expressed in three of the clusters and in four of the samples (Figure 6A–6C). IL-32 was expressed in about 70% of the cells from sample MM33 and at intermediate levels in most cells from MM17 as well as in a few cells from MM36 (Figures 6B and 6C). In patients MM02 IL-32 was not expressed in the bone marrow sample taken at diagnosis (MM02) but highly expressed in all the cells of the extramedullary tumor sample (MM02EM) obtained 18 months later. Importantly, genes involved in “ATP synthesis coupled electron transport,” “assembly of ETC complexes,” and “cell-cycle progression” were significantly upregulated in single cells expressing IL-32 compared with nonexpressing cells (Figure 6D). These data support that the same MM cell that expresses IL-32 has high OXPHOS and proliferation.

### IL-32 expression promotes a more immature plasma cell phenotype

**Figure 7:**
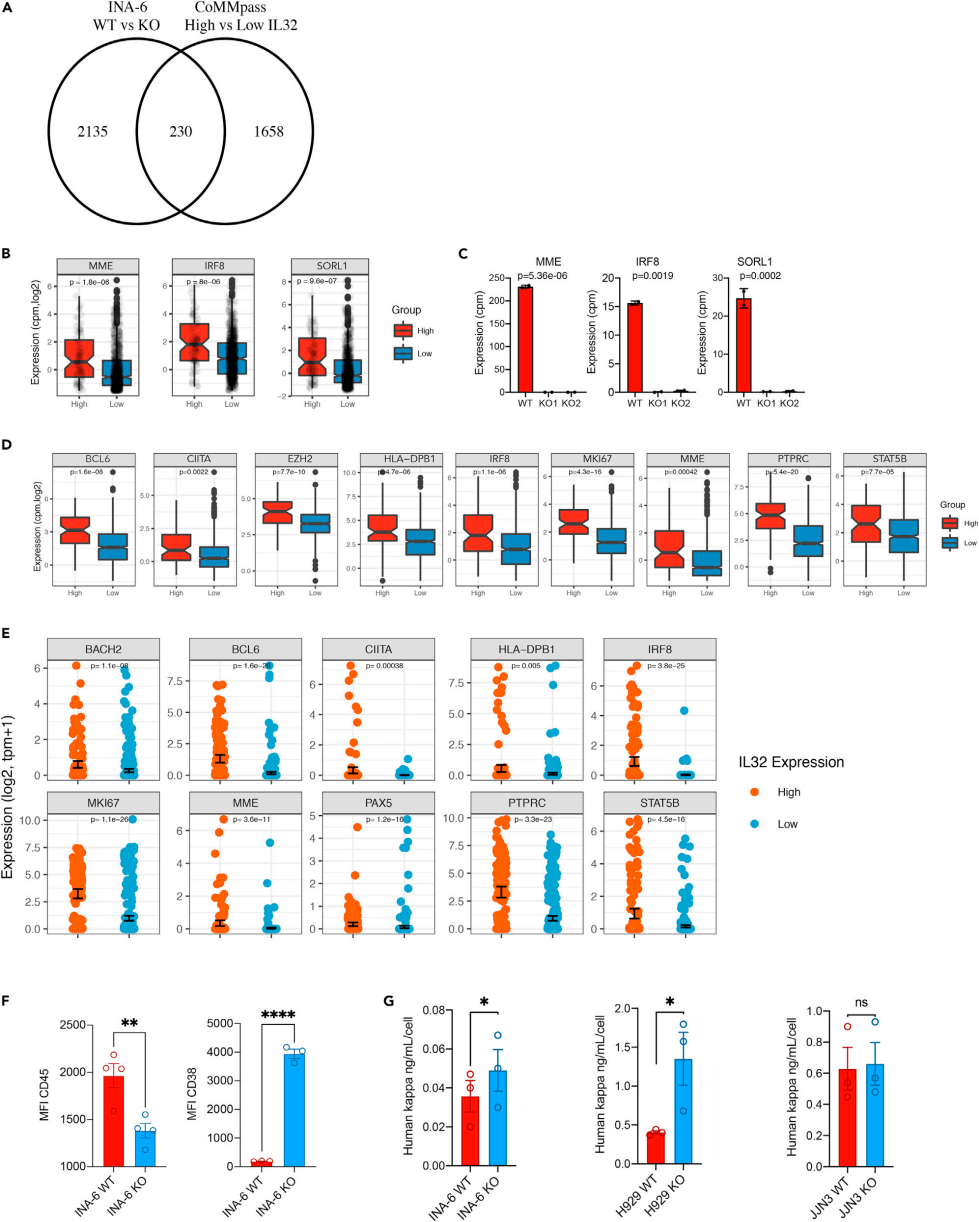
IL-32 expression promotes a more immature plasma cell phenotype (A) Venn-diagram of overlapping significant genes (p <0.01) that were more highly expressed in WT cells compared with KO cells (comparing two INA-6 KO clones [KO1, KO2] with WT mock cells) and upregulated in IL-32 patients (comparing IL-32- expressing patients versus nonexpressing patients). See also Table S5. (B) Gene expression of *MME*, *IRF8*, and *SORL1* in patients expressing IL-32 (10th percentile) compared with nonexpressing (90th percentile) patients. Significance determined by limma in *R*. (C) Gene expression of *MME*, *IRF8*, and *SORL1* in INA-6 IL-32 KO1, KO2, and WT mock cells. Significance determined by limma in *R* with Benjamini-Hochberg-adjusted p-values. Data presented are mean cpm ± SD of two replicates. (D) Evaluation of gene expression of markers associated with less differentiated stages of B cell maturation in CoMMpass IA13, comparing IL-32 expressing patients (upper 10th percentile) with nonexpressing patients (lower 90th percentile). Significance determined by limma in *R*. Boxplots show the median and 25th/75th quantiles and smallest/largest value within the 1.5 times interquartile rang. (E) Scatterplot of genes associated with less differentiated stages of B cell maturation in single cells with (N = 142) and without (N = 346) *IL32*-expression (from single cell transcriptomics). p values were calculated using the FindMarkers function in Seurat by comparing the high and low IL32 groups. (F) Surface expression of CD45 and CD38 in INA-6 KO and WT cells. Data are presented as median fluorescence intensity (MFI) from 3 independent experiments and significance determined by unpaired student's t test. Bare plots show mean ± SEM. (G) Concentration of kappa light chain/cell detected in conditioned media from WT and KO cells as indicated. p values were calculated by the ratio paired t test. ns, not significant; ∗p ≤0.05, ∗∗p ≤0.01, ∗∗∗p ≤0.001, ∗∗∗∗p ≤0.0001.

To gain further knowledge of the transcriptional programs associated with IL-32 in malignant plasma cells, we investigated which genes were more highly expressed in WT compared with INA-6 KO cells and at the same time upregulated in IL-32-expressing primary cells in the CoMMpasss IA13 dataset (Table S5, Figure 7A). We identified 230 genes to be significantly differently expressed in both comparisons, and these genes are likely to be functionally related to IL-32 expression. The top 3 genes, when sorting for the most downregulated genes in KO and upregulated genes in IL-32-expressing patients on the shared signature list, were *MME*, encoding CD10, and the transcription factors *IRF8* and *SORL1*, encoding the sortilin-related receptor 1 (Figures 7B and 7C). SORL1 plays a role in lipid metabolism and IL-6 signaling (Larsen and Petersen, 2017; Mortensen et al., 2014; Patel Kevin et al., 2015), and *MME* and *IRF8* are both important in early stages of B-cell development (Kikuchi et al., 2018; Wang et al., 2008). *MME* and *SORL1* were also downregulated in H929 KO cells compared with WT cells (Figure S7A). *IRF8* was not expressed by this cell line.

Other genes associated with earlier stages of B cell differentiation (Guo et al., 2018; Matthias and Rolink, 2005; Sanz et al., 2019; Scheeren et al., 2005; Vogel et al., 2014; Wilmore et al., 2017), such as *BCL6*, *CIITA*, *EZH2*, *STAT5B*, *PTPRC* (CD45), *MKI67*, and several genes encoding MHC II, including *HLA- DPB1*, were upregulated in IL-32-expressing patients (Figure 7D). Importantly, we also found expression of immature genes to be significantly upregulated in IL-32-expressing cells in the single-cell sequencing dataset (Figure 7E). In accordance, genes associated with mature plasma cells were slightly, but significantly, downregulated in IL-32-expressing patients, including *CD38, CD27*, *CXCR4*, *ERN, PRDM1, IRF4*, and *FOXO1* (Figure S7B). CD45 is as marker of proliferating, immature myeloma cells, whereas CD38 is a marker of terminally differentiated plasma cells and expressed by most MM cells (Paiva et al., 2017). INA-6 is a MM cell line with an immature phenotype, with high expression of CD45 and relatively low expression of CD38. Strikingly, loss of IL-32 led to a reduction in CD45 and re-expression of CD38 in INA-6 cells as examined by flow cytometry (Figure 7F). Other genes associated with immature plasma cells were also downregulated in the KO cells (Figure S7C). ER stress is an Achilles heel of MM cells partly due to the production of large amounts of monoclonal antibodies. IL-32-expressing primary cells seemed less affected by ER stress (Figure 5E), so we asked whether this could be related to less production of immunoglobulins. Strikingly, INA-6 and H929 KO cells secreted more kappa light chain than WT mock cells (Figure 7G), supporting our hypothesis that IL-32 expression promotes an immature plasma cell phenotype and that IL-32-expression may relieve the cells from immunoglobulin-related ER stress. Taken together these results suggest that IL-32 is involved in regulation of transcriptional programs that induce a more immature and less ER-stressed plasma cell.

## Discussion

We have identified IL-32 as a novel, endogenously expressed growth and survival factor for malignant plasma cells. IL-32 interacts with components of the respiratory chain, and expression of IL-32 is important for efficient OXPHOS in MM cells. A subgroup of MM patients expresses IL-32, and these patients have reduced OS. Furthermore, the malignant plasma cells of these patients have distinct phenotypical characteristics, resembling an immature or less differentiated plasma cell.

Based on gene expression data in CoMMpass, 10% to 15% of MM patients express IL-32 at diagnosis. Moreover, analyses of paired samples from diagnosis and relapse in individual patients suggest that IL-32 expression increases upon relapse in about 20% of the patients. The strong, independent association of IL-32 with inferior survival in patients, the reduced proliferative rate, and the reduction of OXPHOS in three phenotypically different cell lines when depleting IL-32 strongly suggest that IL-32 plays a role in MM disease progression. The IL-6-dependent cell line INA-6 was most dependent on IL-32 expression, because loss of IL-32 reduced not only proliferation but also cell survival. In fact, these cells were not able to form tumors *in vivo*, even in a supportive, humanized bone marrow microenvironment. This can possibly be explained by IL-32-depleted cells being less able to adapt to the more challenging metabolic conditions *in vivo*, where there may be limited access to oxygen and changed composition of nutrients (Muir et al., 2018; Sullivan et al., 2019). It is also worth noting that IL-32 is expressed by regulatory or senescent/exhausted T cells in MM, although if/how this affects the function of the T-cells and if it contributes to disease progression is not known (Bailur et al., 2019; Zavidij et al., 2020).

In contrast to most MM cells, which in general are slow growing and have low Ki-67, (Gastinne et al., 2007; Wilson et al., 2001), IL-32-expressing primary MM cells have a gene signature related to cell division and an immature plasma cell phenotype. This was evident both from the large CoMMPass dataset and from the small number of single-cell RNA-sequenced patient samples. And strikingly, IL-32-depleted MM cells had downregulated expression of the same “immaturity” genes as compared with WT cells. We cannot conclude whether the changes in proliferation and gene expression are related to the metabolic effects of IL-32 or an independent effect of IL-32 on transcription. Metabolites may, however, play a central role in regulating gene expression. For example, availability of acetyl-CoA can modify extent of histone acetylation, whereas metabolites such as succinate and a-ketoglutarate may regulate DNA and histone methylation (van der Knaap and Verrijzer, 2016). Although additional experiments are needed to conclude, it could be that IL-32 expression and subsequent changes in metabolism may lead to plasma cell de-differentiation or maturation arrest. The profound changes in ATP and core metabolites such as citrate, a-ketoglutarate, succinate, fumarate, and malate upon IL-32 depletion as shown here makes this a possible scenario. Supporting this notion, GO terms related to histone modifications were also differently expressed between IL-32-expressing and nonexpressing primary cells. Of note, MM cells with an immature phenotype have previously been linked with more aggressive tumors (Leung-Hagesteijn et al., 2013; Paiva et al., 2017) and compounds such as ATRA and 2-methoxyestrodiol (2-ME2) that promote differentiation of MM cells render the cells more sensitive to bortezomib (Gu et al., 2012). Thus, IL-32 could potentially be a marker for patients that may benefit from such combined treatment.

Oxygen is a key regulator of aerobic respiration and metabolism, and it is striking that IL-32 expression is regulated by two different oxygen sensing systems, HIF1α (Zahoor et al., 2017) and cysteamine (2-aminoethanethiol) dioxygenase (ADO) (Masson et al., 2019). ADO is an enzymatic O_2_ sensor and was shown to catalyze dioxygenation of IL-32 in the presence of O_2_, leading to proteasomal degradation (Masson et al., 2019). Correspondingly, hypoxia leads to stabilization of HIF1α, which induces IL-32 mRNA and protein expression (Zahoor et al., 2017). These data support that IL-32 has an important role in cellular responses to alterations in oxygen levels. Hypoxia is known to cause changes in the composition of ETC complexes and the changes help keep the mitochondria intact under low oxygen conditions and to prevent excessive ROS formation (Fuhrmann and Brüne, 2017). Indeed, we found that 7 of 33 proteins that bound to IL-32 in hypoxia were located in the mitochondrion and that 5 of these were subunits of different components of the mitochondrial respiratory chain. Respirasome supercomplexes, where the respiratory chain components are assembled in close vicinity to each other, lead to higher catalytic activity of the individual components, to increased efficiency of electron transfer, and to less production of ROS (Guo et al., 2016; Lenaz and Genova, 2012). The IL-32 KO cells had reduced capacity for mitochondrial respiration and ATP formation; still, cells lacking IL-32 had significantly higher levels of mitochondrial ROS, in line with suboptimal respiration. Thus, we propose that IL-32 by binding components of the respiratory chain enhances the efficiency of the ETC, enabling the cells to maintain OXPHOS even under conditions of low O_2_ and also to keep mtROS at a level compatible with cell survival. Exactly how IL-32 is transported to and acts in the mitochondria to enhance OXPHOS needs to be further explored.

IL-32-depleted cells had dramatic alterations in the composition of lipids with a profound accumulation of unsaturated TAG. This could be due to reduced oxidation in the electron transport chain, leading to enhanced transport of citrate out from the mitochondria and used for synthesis of fats (Moen et al., 2016). It is also possible that the accumulation of neutral fats is a result of cellular stress (Petan et al., 2018). A third possibility is that IL-32 plays a more direct role in lipid metabolism. For example, we found that SORL1 was highly expressed in IL-32-expressing patients and downregulated in the IL-32 KO cells. SORL1 encodes the sortilin-related receptor 1, a multifunctional intracellular sorting protein belonging to the sortilin and LDL-receptor families implicated in the regulation of intracellular lipid pools (Klinger et al., 2011). How IL-32 acts at the molecular level to regulate lipid synthesis, lipid metabolism, or lipid transport is however unclear.

In conclusion, we have shown that intracellular IL-32 promotes OXPHOS and provides a survival benefit for malignant plasma cells. The interaction of IL-32 with components of the respiratory chain and its regulation by two different oxygen sensing system indicate that IL-32 has an important role in cellular responses to O_2_ fluctuations. Besides identifying IL-32 as a potential prognostic biomarker and treatment target in MM, our results provide insight into the metabolic functions of IL-32, which may be further exploited in other cancers and inflammatory diseases where IL-32 is known to play a central role.

### Limitations of the study

Our *in vitro* findings from cell lines on the importance of IL-32 for proliferation, OXPHOS, and plasma cell maturation are supported by gene expression analyses of primary cells from patients. Here we observe that IL-32-expressing myeloma cells have a gene signature indicative of highly proliferating cells with active OXPHOS and an immature phenotype. Ideally, we should have verified the link between OXPHOS, proliferation, and IL-32 expression in patient samples by genetic manipulation of primary plasma cells. However, that is challenging because primary myeloma cells have very poor viability *ex vivo*. We also demonstrate that IL-32 binds to components of the mitochondria electron transport chain, and we hypothesize that IL-32 may increase the efficiency of the electron transport chain by direct protein interactions. However, how this happens at the molecular level needs to be investigated further. The link between IL-32 expression and plasma cell maturation could be strengthened by performing flow cytometric analyses of bone marrow aspirates from patients. Such experiments would however require access to freshly obtained bone marrow aspirates from a large number of patients.

## STAR★Methods

### Key resources table

**Table:** 

REAGENT or RESOURCE	SOURCE	IDENTIFIER
**Antibodies**		

CD38 PE-Cy^TM^7	BD Biosciences	#335825 (clone HB7); RRID:AB_2868688)
FITC Mouse anti-human CD45	BD Biosciences	#555482(clone HI30); RRID:AB_395874
β-actin antibody	Cell Signaling Technology	#4967; RRID:AB_330288
Human IL-32 antibody	R&D Systems	#AF3040; RRID:AB_2124022
β-Tubulin (9F3) Rabbit mAb	Cell Signaling Technology	#2128; RRID:AB_823664
COX IV (3E11) Rabbit mAb	Cell Signaling Technology	#4850; RRID:AB_2085424
Anti-TOMM20 antibody produced in rabbit	Sigma Aldrich	#HPA011562; RRID:AB_1080326
Anti-NDUFA12 antibody produced in rabbit	Sigma Aldrich	#HPA039903; RRID:AB_10795516
Anti-GAPDH antibody(6C5)	Abcam	#ab8245; RRID:AB_2107448
Anti-ATP5D antibody	Abcam	#ab97491; RRID:AB_10681010
anti-ATP synthase immunocapture antibody (12F4AD8AF8)	Abcam	#ab109867; RRID:AB_10866627
Alexa Fluor® 488 Anti-BrdU antibody [BU1/75 (ICR1)]	Abcam	#ab220074
Chicken anti-goat IgG (H+L) Cross-adsorbed Secondary Antibody Alexa Fluor 647	Thermo Fisher	# A21469; RRID:AB_1500603
Donkey anti-Rabbit IgG (H+L) Highly Cross-Adsorbed Secondary Antibody Alexa Fluor 488	Thermo Fisher	#A21206; RRID:AB_2535792

**Chemicals, peptides, and recombinant proteins**		

Recombinant human IL-32 gamma protein/CF	R&D Systems	#6769-IL-025
Recombinant human IL-32 gamma protein/CR	R&D Systems	#4690-IL-025/CF
Oligomycin from Streptomyces diastatochromogenes	Merck	#O4876
2-deoxy-d-glucose	Sigma Aldrich	# D8375
Rotenone	Sigma Aldrich	#R8875
Antimycin A1	Sigma Aldrich	#A0149
FCCP (Carbonyl cyanide 4-(trifluoromethoxy)phenylhydrazone)	Sigma Aldrich	#C2920
Nile Red	Thermo Fisher	#N1142
BrdU (5-bromo-2'-deoxyuridine), Thymidine analog	Abcam	#ab142567
eBioscience™ Fixable Viability Dye eFluor™ 450	Thermo Fisher	#65-0863-14
Hoechst 33342 solution	Thermo Fisher	#62249
MitoTracker® Orange CMTMRos	Thermo Fisher	#M7510
Tetramethylrhodamine, methyl ester (TMRM)	Thermo Fisher	# T668
MitoTracker Green FM	Thermo Fisher	#M7514
IACS-10759	Axon Medhcem	#Axon2909

**Critical commercial assays**		

Human Kappa ELISA Kit	Bethyl Laboratories	#E88-115
MitoSOX^TM^ Red Mitochondrial Superoxide Indicator	Thermo Fisher	#M36008
Corning™ Cell-Tak Cell and Tissue Adhesive	Fisher Scientific	#10317081
Seahorse XFe96 FluxPak and Seahorse XF base medium	Agilent	# 102416-100 # 102353-100
CellTiter-Glo® Luminescent Cell Viability Assa	Promega	#G7570
Lonza™ Cell Line Nucleofector™ Kit R	Lonza	#VCA-1001
Dynabeads™ Antibody Coupling Kit	Thermo Fisher	#14311D
Apoptest Annexin A5- FITC kit	VPS Diagnostics	#A700
TruSeq Stranded mRNA library preparation kit	Illumina	#20020595

**Deposited data**		

Metabolomics INA-6 KO and WT cells	Mendeley Data	10.17632/dyndvz5vfn.1
RNA-seq INA-6 KO and WT cells	SRNA	PRJNA769223
Raw images for all western blots	Mendeley Data	10.17632/hcdmjrjhft.1
R-codes	Github	https://github.com/MjelleLab/IL32.git

**Experimental models: Cell lines**		

INA-6	CLPUB00090	RRID:CVCL_5209
NCI-H929	ATCC	#CRL-9068
JJN-3	(Hamilton et al., 1991)	RRID: CVCL-2078

**Experimental models: Organisms/strains**		

RAG2^−/−^ γC^−/−^ BALB/c	(Groen et al., 2012)	N/A

**Oligonucleotides**		

IL32 (Hs00992441_m1)	Thermo Fisher	# 4331182
TBP (Hs00427620_m1)	Thermo Fisher	# 4331182

**Recombinant DNA**		

pLV-U6-IL32/EF1a-puro-2A-CAs9-2A-GFP.	Sigma-Aldrich	# HS0000050421
Sigma lenti CRISPR Non-Targeting Control	Sigma-Aldrich	CRISPR12V-1EA
PsPAX2	Gift from Didier Trono (unpublished)	Addgene plasmid #12260
pMD2.G	Gift from Didier Trono (unpublished)	Addgene plasmid #12259
IL-32 CRISPR/Cas9 KO plasmid (h2)	Santa Cruz	#sc-406489-KO-2
OmicsLink™ ORF lentiviral IL-32β vector	GeneCopoeia	#EX-M0733-Lv121
OmicsLink™ ORF lentiviral control vector	GeneCopoeia	#EX-EGFP-Lv121
pIRFP	(Filonov et al., 2011)	Addgene plasmid #31857

**Software and algorithms**		

Image Studio Software version 3.1	LI-COR	https://www.licor.com/bio/image-studio/resources RRID: SCR_015795
Fiji	(Schindelin et al., 2012)	https://imagej.net/software/fiji/RRID: SCR_002285
FlowJo v10.7	FlowJo	https://www.flowjo.com/solutions/flowjo RRID: SCR_008520
GraphPad Prism software	GraphPad	RRID: SCR_002798
R-studio software	RStudio	RRID: SCR_000432
Leica Application Suite X 3.55.19976	Leica Microsystems	RRID: SCR_013673

**Other**		

Corning™ Cell-Tak Cell and Tissue Adhesive	Fisher Scientific	#10317081

### Resource availability

#### Lead contact

Further information and requests for resources and reagents should be directed to the lead contact Therese Standal (therese.standal@ntnu.no).

#### Materials availability

This study did not generate new unique reagents.

### Experimental model and subject details

#### *In vivo* animal studies

RAG2^−/−^ γC^−/−^ BALB/c were obtained from Dr. Anton Martens (University Medical Center, Utrecht, the Netherlands) (Groen et al., 2012). The mice lack B, T and NK cell immunity and were kept in specific pathogen free (SPF) unit. The mice were housed in IVC-cages, with free access to bedding material, nesting material and enrichment objects. Mice were given sterile food (RM1 #801002, Special Diets Services, Essex, UK) and water ad libitum, and were caged in groups of 3 to 5 mice. The mice were kept at a room temperature of 21°C to 22°C and 55% humidity with a 12-h light/dark cycles including 1 hour dusk/dawn.

For the INA-6 *in vivo* experiment 20 female mice RAG2^−/−^ γC^−/−^ BALB/c (12–16 weeks old) were used. Human bone marrow-derived mesenchymal stromal cells (hMSC) from healthy donors were seeded on biphasic calcium phosphate (BCP) scaffolds and differentiated toward osteoblasts for 1 week *in vitro* before implanted subcutaneously on the back of the mice (4 scaffold on each mice) and left for 2 months for establishment of a differentiated human bone cell microenvironment as described (Groen et al., 2012). 3 scaffolds on each mouse were injected with 10ˆ6 iRFP labelled INA-6 KO or WT cells and imaging was performed after injection (timepoint 0) and then every week for 5 weeks. Images were acquired at 700 nm using Pearl Impulse imaging system and data was analyzed with Image Studio Software (both from Licor Biosciences). iRFP signal from each scaffold on each time point was normalized to the signal at day 0. For the JJN3 *in vivo* experiment JJN3 1 × 10^5^ IL-32 KO (N = 5) or IL-32 WT (N = 5) cells were injected into the tibiae of 9 to 12-week-old RAG2^−/−^ γC^−/−^ BALB/c. After 20 days, the mice were euthanized. Blood was collected for quantification of human immunoglobulin kappa light chain by ELISA (Bethyl Laboratories). Animal handling and procedures were approved by the Norwegian food safety authority (FOTS10517).

#### Cell culture

MM cell lines INA-6 and JJN-3 were kind gifts from Dr. Martin Gramatzki (University of Erlangen-Nuremberg, Erlangen, Germany), and Dr. Jennifer Ball (University of Birmingham, UK), respectively. NIC-H929 cells (named H929 throughout the paper) were obtained from ATCC. H929 and JJN-3 were cultured in 10% heat inactivated fetal calf serum (FCS) in RPMI-1640 (RPMI) medium. INA-6 cells were cultured in 10% FCS in RPMI with the addition of 1 ng/mL recombinant human (rh) interleukin (IL)-6. MM cell lines were maintained at 37°C in a humidified atmosphere with 5% CO_2_. HEK293T cells (Open Biosystems, Thermo Fisher Scientific), were cultured in 37°C in a humidified atmosphere with 8% CO_2_ in DMEM supplemented with 10% FCS.

#### Generation of IL-32-depleted cells

IL-32 KO cell lines were generated by lentiviral transduction (H929 cells) or plasmid transfection (JJN-3 and INA-6 cells). H929 cells were transduced using lentiviral CRISPR IL-32 KO vector pLV-U6-gRNA/EF1a-puro-2A-CAs9-2A-GFP and Sigma lenti CRISPR Non-Targeting Control virus particles. The IL-32 KO vector were packaged into viral particles in HEK293T cells using second generation packaging plasmids psPAX2, and pMD2.G. After 48 h, virus particles in supernatants were transduced into target cells incubated with polybrene (8 μ g/mL). Ready to use control virus particles were delivered to the cells by spin transduction with the same concentration of polybrene. The H929 IL-32 KO and control vector cells (H929 WT) were then subjected to negative selection using puromycin (0.5 μ g/mL) followed by single cell cloning. JJN-3 and INA-6 cells were electroporated with IL-32 CRISPR/Cas9 KO plasmid containing green fluorescent protein (GFP) for selection, using buffer R (Amaxa Nucleofector Kit R, Lonza) and program R-001. Cells were then sorted for GFP positivity on a FACSAria Fusion flow cytometer (BD Biosciences) and single cell cloned. Clones were screened for IL-32 expression by flow cytometry and immunoblotting. For both cell lines: clones that had been transfected with the plasmid (GFP^+^), but still expressed IL-32 were used as control cells (IL-32 wild type [WT]). IL-32 protein knockout was regularly confirmed by immunoblotting to ensure homogeneity of the KO cell lines.

#### Generation of IL-32 knock-in cells

INA-6 IL-32 knock in cells were generated by lentiviral transduction of INA-6 IL-32 KO using OmicsLink ORF lentiviral expression system with vector for knock in of IL-32β (transcript variant 8) EX-M0733-Lv121 and control vector: EX-EGFP-Lv121. Lentiviral packaging and transduction of cells were performed as described for IL-32 depletion of H929 followed by puromycin selection.

#### Generation of iRFP-labelled cells

iRFP labelling of INA-6 KO and WT cells was performed by lentiviral transduction. For generation of iRFP plasmid, piRFP and pENTR4 (Invitrogen) were cut with SalI and NotI (Fermentas) and ligated using a T4 DNA ligase (Fermentas) to achieve a gateway compatible iRFP entry clone. The iRFP-ENTR4 was then recombinated into the pLenti-CMV-Puro-Dest by a LR (Invitrogen) gateway reaction. iRFP lentivirus was made by transfecting HEK293T using iRFP plasmid, psPAX2 and pMD2.G packaging plasmids followed by transduction of target cells with polybrene. After transduction, iRFP-positive cells were sorted by fluorescence-activated cell sorting giving a pure iRFP-labelled population for *in vivo* studies.

### Method details

#### Assessment of proliferation and survival

Proliferation of IL-32 KO and WT mock cells was assessed by cell counting every day for 4 days using cell coulter (Beckman Coulter Diagnostics). Proliferation was also examined by brdu(5-bromo-2′-deoxyuridine) incorporation to quantify % of live cells in S-phase cell cycle progression.

Brdu incorporation was performed with the following procedure:

The cells were seeded out at a concentration of 333,000 cells/mL and incubated for 4 h with 10 μM BrdU in the culture media. The cells were washed once in PBS and subsequently stained with Fixable Viability Dye eFluor 450 for 30 min on ice. The cells were washed in PBS and thereafter fixed in methanol. The cells were stored in methanol at 4°C for up to three days before they were spun down and resuspended in PBS before vortexed while slowly adding 1 mL of 2N HCl/0.05 %Triton x-100 to denature the DNA. The cells were then incubated at room temperature for 30 min followed by centrifugation to remove supernatant. The cells were then resuspended in 1 mL of 0.1 M Na2B4O7 (Borax), pH 8.5 for neutralization before the cells were spun down to remove the supernatant. Cells were then stained with Alexa Fluor 488 Anti-BrdU in PBS with 1% BSA/0.1% Tween and analyzed using LSRII FACS Flowcytometer (BD, USA). Gating of live cells, single cells and quantification of brdu + cells were performed with Flow Jo version 10.

Cell viability was assessed with Annexin/PI flow cytometry. Cells were sampled directly from cell culture flasks, stained with Apoptest Annexin A5- FITC kit following manufacturer's instructions and analyzed by flow cytometry using LSR II (BD Biosciences) with FACS Diva software (BD Biosciences).

For experiments using the OXPHOS inhibitor IACS-10759 INA-6, H929 and JJN-3 IL-32 KO and WT mock cells were seeded at a density of 33,000 cells/mL and grown in medium supplemented with IACS-10759 (10 nM). Number of cells was determined by counting after 4 days of culture using cell coulter (Beckman Coulter Diagnostics).

To examine the effect of rhIL-32 *β* and rhIL-32 y on cell proliferation and viability, the cells were treated with 100 ng/mL recombinant IL-32 (R&D Systems). Proliferation was assessed either by cell counting using cell coulter (Beckman Coulter Diagnostics) or by CTG assay (Promega). For cell counting experiments cells were counted every day for 3 days. For CTG assay 2500 cells/well were seeded in 96-well optical plates and signal measured every day for 3 days. Data was normalized to the luminescent signal from day 0 when cells were seeded in equal number. Viability after treatment with rhIL-32 isoforms *β* and *γ* overnight was evaluated by trypan blue staining.

#### Real-time quantitative PCR

Total RNA was isolated using RNeasy Mini Kit (Qiagen) and complementary DNA (cDNA) was synthesized from total RNA using the High Capacity RNA-to-cDNA kit (Applied Biosystems). qPCR was performed using StepOne Real-Time PCR System and Taqman Gene Expression Assays (Applied Biosystems) with standard settings (2′ 50°C, 10′ 95°C, 40 cycles at 95°C for 15 sec, 1′ 60°C). The comparative Ct method was used to estimate relative changes in IL-32 gene expression using TBP as housekeeping gene. Probes were as follows: human IL-32 (Hs00992441_m1) housekeeping gene TATA binding protein (*TBP*; Hs00427620_m1)

#### Immunoblotting

Cells were lysed in lysis buffer (50 mM Tris–HCl, 1% NP40, 150 mM NaCl, 10% glycerol, 1 mM Na_3_VO_4_, 50 mM NaF and Complete protease inhibitor (Roche Diagnostics) and the lysates were denatured in 1× NuPage LDS sample buffer supplemented with 0.1 mM DTT for 10 min at 70°C before they were separated on 10% Bis-Tris polyacrylamide gel. Proteins were transferred to a nitrocellulose membrane using the iBlot Dry Blotting System (Invitrogen). Membranes were blocked using 5% bovine serum albumin (Sigma–Aldrich) in Tris-buffered saline with 0.01% Tween followed by overnight incubation with the primary antibodies previously described. Detection was performed using horseradish peroxidase (HRP) conjugated antibodies (DAKO) and developed with Super Signal West Femto Maximum Sensitivity Substrate (Thermo Scientific). Images were obtained with LI-COR Odyssey Fc and analyzed using Image Studio Software (LI-COR).

#### Surface markers

For assessment of CD38 and CD45 on IL-32 KO and WT cells, live cells were stained with anti- CD38 PE-Cy7 and CD45-FITC antibodies and assessed by flow cytometry. Samples were analyzed with FlowJo V10.

#### ELISA

For confirmation of biological activity of recombinant human recombinant IL-32, peripheral blood mononuclear cells (PBMCs) were isolated from blood from healthy human donors were provided by the blood bank as St. Olavs Hospital (REK#2009/2245) with a density gradient using Lympoprep (Alere). CD14^+^ cells were isolated from the PBMCs using CD14 magnetic beads (CD14 MicroBeads UltraPure, Human, Miltenyi Biotech). The CD14^+^ cells were seeded out 30,000 cells/well in a 96-well plate in RPMI 1640 (Sigma) supplemented with 10% human serum, 10mM HEPES (Gibco) and 10 ng/mL M-CSF (R&D Systems). At day 3, half the medium was changed. At day 6, the cells were rested in medium without M-CSF. At day 8, the cells were stimulated with 100 ng/mL rhIL-32 beta and IL-32 gamma in RPMI 1640 (Sigma) supplemented with 2% FCS, 10mM HEPES (Gibco). After 24 h of stimulation the supernatant was harvested. TNF a in the supernatant was quantified using Human TNF-alpha DuoSet ELISA (R&D systems) according to the manufacturer's instructions.

For quantification of immunoglobulin kappa light chain secretion from myeloma cell lines 1∗10^6^ cells were seeded in 1 mL RPMI, 0.1% BSA with the addition of 1 ng/mL IL-6 for INA-6 cells and incubated for 24 h in 5% O_2,_ 37°C. The cells were counted, and Ig kappa light chain concentration were quantified in the cell culture supernatant by ELISA (Human Kappa ELISA, Bethyl Laboratories).

#### Co-immunoprecipitation and mass spectrometry

IL-32 antibody (RnD) was conjugated to M-270 beads according to the manufacturer's instructions, using Dynabeads Antibody Coupling Kit and 10 mg beads on 500 μL (100ug) resuspended IL-32 antibody. For CO-IP MS analysis JJN-3 KO and WT cells were incubated in hypoxia for 24 h before lysis with 4x pellet volume RIPA buffer (1% CHAPS, 50 mM Tris, 150 mM NaCl, Complete protease inhibitor (Roche Diagnostics) and phosphatase inhibitor cocktails 2 and 3 (Sigma-Aldrich), for 1 h at 4°C on rotation. Lysate was pre-cleared by adding 40μL beads to ∼400 μL lysate for 1 h at 4° on rotation, before incubated with IL-32 antibody conjugated beads for 2 h at 4°C. After washing 4 times with PBS repelleted beads were subjected to MS-digestion protocol. Beads were resuspended 150 μL 50 mM NH4HCO3, followed by addition of 7.5 μL 200 mM DTT, 55°C for 30 min. Samples were cooled to RT before addition of 15 μL 200mM IAA and incubation in RT for 30 min in the dark. Then samples were treated 1.5 μg trypsin (MS-grade) at 37°C over-night, before beads were removed and liquid sample was dried using Speedvac (Thermo Fischer Scientific).

After tryptic digestion peptides were desalted using STAGETIP as previously described (Rappsilber et al., 2003). After desalting, peptides were dried down in a SpeedVac centrifuge and resuspended in 0.1% formic acid. The peptides were analyzed on a LC-MS/MS platform consisting of an Easy-nLC 1200 UHPLC system (Thermo Fisher Scientific) interfaced with an QExactive HF orbitrap mass spectrometer (Thermo Fisher Scientific) via a nanospray ESI ion source (Proxeon). Peptides were injected into a C-18 trap column (Acclaim PepMap100, 75 μm i. d. × 2 cm, C18, 3 μm, 100 Å, Thermo Fisher Scientific) and further separated on a C-18 analytical column (Acclaim PepMap100, 75 μm i. d. × 50 cm, C18, 2 μm, 100 Å, Thermo Fisher Scientific) using a multistep gradient with buffer A (0.1% formic acid) and buffer B (80% CH3CN, 0.1% formic acid): From 2% to 10% B in 10 min, 10% to 50% B in 130 min, 50% to 100% B in 20 min and 20 min with 100% buffer B. The HPLC were re-equilibrated with 2% buffer B before next injection. The flow rate was 250 nL/min. Peptides eluted were analyzed on QExactive HF mass spectrometer operating in positive ion- and data dependent acquisition mode using the following parameters: Electrospray voltage 1.9 kV, HCD fragmentation with normalized collision energy 32, automatic gain control target value of 3E6 for Orbitrap MS and 1E5 for MS/MS scans. Each MS scan (m/z 300–1600) was acquired at a resolution of 12,000 FWHM, followed by 15 MS/MS scans triggered for AGC targets above 2E3, at a maximum ion injection time of 50 ms for MS and 100 ms for MS/MS scans.

5 replicates each of KO and WT cells were used for mass spectrometry analysis. The IL32-specific proteins were detected by subtracting the peptides detected in the KO-cells from the peptides detected in the WT-cells. We required the peptides to be detected in all 5 WT replicates. To assess the probability of detecting a frequency of 7 mitochondrial out of 36 IP target proteins we based the expected frequency of 1100 mitochondrial proteins (Calvo and Mootha, 2010) and 20,000 proteins as the total number of proteins in the human proteome.

For validation of interaction partners in the mitochondrial electron transport chain we chose two candidates for validation, ATP synthase subunit delta (ATP5D) a subunit in the ATP-synthase complex (complex IV) and NADH dehydrogenase [ubiquinone] 1 alpha subcomplex subunit 12 (NDUFA12), a subunit of the NADPH dehydrogenase (complex I). IL-32 was pulled down following the same protocol as earlier, in INA-6, IH-1 and JJN-3 cells, and ATP5D and NDUFA12 were detected by western blotting.

#### Metabolomics

Targeted metabolomics (GC-MS and LS-MS) of INA-6 WT and two KO cell lines were performed by MetaSysX (Potsdam-Golm, Germany). The sample preparation was performed according to the company's standard procedure, a modified protocol from Salem et al. (Salem et al., 2016). 10ˆ8 cells/replicate was used for metabolite extraction.

LC-MS Measurements (Hydrophilic and Lipophilic Analytes) were performed using Waters ACQUITY Reversed Phase Ultra Performance Liquid Chromatography (RP-UPLC) coupled to a Thermo-Fisher Exactive mass spectrometer. C8 and C18 columns were used for the lipophilic and the hydrophilic measurements, respectively. Chromatograms were recorded in Full Scan MS mode (Mass Range [100–1500]). All mass spectra were acquired in positive and negative ionization modes. Extraction of the LC-MS data was accomplished with the software REFINER MS 11.1 (GeneData, http://www.genedata.com). Alignment and filtration of the LC-MS data were completed using metaSysX in-house software. After extraction from the chromatograms, the data was processed, aligned and filtered for redundant peaks. The alignment of the extracted data from each chromatogram was performed according to the criteria that a feature had to be present in at least 3 out of 4 replicates from one group. At this stage, the average RT and *m/z* values was given to the features. The alignment was performed for each type of measurement independently, followed by the application of various filters in order to refine the dataset, which included the removal of isotopic peaks, in-source fragments of the analytes (due to the ionization method), and redundant peaks like additional less intense adducts of the same analyte and redundant derivatives, to guarantee the quality of the data for further statistical analyses. The in-house metaSysX annotation database of chemical compounds was used to match features detected in the LC-MS polar and lipophilic platform. The annotation of the content of the sample was performed by database query of mass-to-charge ratio and the retention time of detected features within certain criteria.

The metaSysX in-house database contains mass-to-charge ratio and retention time information of 7500 reference compounds available as pure compounds and measured in the same chromatographic and spectrometric conditions as the measured samples. In addition, 1500 lipids and sugar esters were putatively annotated based on the precursor m/z, fragmentation spectrum and elution patterns. The matching criteria for the polar and non-polar platforms were 5 ppm and 0.085 min' _deviation from the reference compounds mass-to-charge ratio and retention time, respectively. Coeluting compounds with the same mass-to-charge ratio were all kept and the names are separated with "|". Lipid annotation was additionally performed and confirmed by MS/MS fragmentation spectrum using the metaSysX developed-R-based algorithm. This information is combined with the information from the annotation after the query of the MSX database.

GC-MS measurements were performed on an *Agilent Technologies* GC coupled to a *Leco Pegasus HT* mass spectrometer which consists of an EI ionization source and a TOF mass analyzer (column: 30 meters DB35; Starting temp: 85°C for 2min; Gradient: 15°C per min up to 360°C). NetCDF files that were exported from the Leco Pegasus software were imported to "R". The Bioconductor package TargetSearch (Cuadros-Inostroza et al., 2009) was used to transform retention time to retention index (RI), to align the chromatograms, to extract the peaks, and to annotate them by comparing the spectra and the RI to the Fiehn Library and to a user created library. The annotation of peaks was manually confirmed in Leco Pegasus. Analytes were quantified using a unique mass. Metabolites with an RT and a mass spectra that did not result in a match in the database were kept as not assigned metabolites. Statistical analysis of significantly upregulated and downregulated metabolites in INA-6 KO clones compared to WT, metabolite enrichment, and joint pathway analysis for metabolomics and transcriptomics (RNA-seq) data was performed using Metaboanalyst 4.0 software. Specifically, in Metaboanalyst 4.0, normalized metabolite values with Peak IDs as provided by metaSysX were uploaded together with significant gene symbols (Fold change >0.5 or < −0.5 and adjusted p value <0.05) with corresponding logFC values from the INA-6 cell line. The significant genes for the INA-6 cell line were determined using *limma* in R using the script provided in the section “Data and code availability”.

#### Confocal imaging

For IL-32/mitochondria colocalization and mitochondrial morphology studies cells were cultured in hypoxia (2% O_2_, 5% CO_2_) overnight before seeded in poly-L-lysine coated 96 well glass bottom plates (In Vitro Scientific) and left to attach for 20 min at 37°C before fixed with 4% PFA, 10 min at RT. After quenching for 10 min with 50 mM NH_4_Cl, permeabilization was performed using 0.05% saponin in PBS. Primary antibody cocktail (anti-IL-32 and anti-TOMM20 or anti-TOMM20 only, 2 μg/mL) diluted in 1% HS, 0.05% saponin was left on overnight at 4°C. Next day, secondary antibody (Donkey anti-Rabbit IgG (H + L) Alexa Fluor 488 or/and Chicken anti-goat IgG (H + L) Antibody Alexa Fluor 647, 2 μg/mL) in 1% HS, 0.05% saponin was added for 30 min, before leaving cells in Hoechst (Thermo Fisher) in PBS (2 μg/mL) for imaging.

For lipid droplet staining with Nile Red cells were attached to poly-L-lysine coated 96-well glass bottom plates, before fixed with 4% PFA, 10 min at RT and stained with 500 nM Nile Red (Thermo Fisher) for 10 min at 37°C. Polar lipids were excited at 590 nm (600–700 nm) and neutral lipids at 488 nm (500–580 nm) as described previously (Schnitzler et al., 2017).

All confocal imaging was performed on a Leica TCS SP8 STED 3X confocal laser scanning microscope (Leica Microsystems, Wetzlar, Germany) using a 63×/1.40 oil objective. Images of mitochondria (colocalization and morphology) were deconvoluted using Huygens Professional software (Hilversum, the Netherlands).

Quantification of mitochondrial length was performed in Fiji Software, using ROI manager to measure the length of each individual mitochondria in 3 cells/image in 5 different images for each independent experiment. Mean length of mitochondria in IL-32 KO and WT mock cell lines was calculated based on 3 independent experiments.

Quantification of the colocalization between IL-32 and mitochondria was performed using Leica Application Suite X, with a threshold for colocalization and background at 30% on both IL-32 and TOMM20 channels. With this settings, colocalization rate (CR) were analyzed for 4 images with 1 to 3 cells/image from each of the cell lines (hypoxic H929, INA-6 and JJN-3 WT cells) and mean CR±SD were calculated. The colocalization rate value is the ratio of the area of colocalizing fluorescence signals and the area of the image foreground.

#### Seahorse metabolic assays on cells

Oxygen consumption rates (OCR) and extracellular acidification rates (ECAR) were measured using Seahorse XF96 bioscience extracellular flux analyzer (Agilent). Seahorse XF Cell culture microplates were treated with Cell Tak according to the manufacturer's instructions and number of viable cells was determined using Countess Automated cell coulter with trypan blue stain before plated in 22 or more replicates at a density of 25,000 cells/well. For measurement of basal and maximal OCR the cells were analyzed by mito stress assay. For mito stress assay cells were incubated in XF assay base medium supplemented with 10 mM Na-Pyruvate, 2 mM glutamine (both from Sigma) and 10 mM glucose (Merck) followed by injections of oligomycin, carbonyl cyanide p-trifluoro-methoxyphenyl hydrazone (FCCP); and antimycin A+ rotenone at final concentrations of 1 μ M, 1 μ M, and 2μ M + 2 μ M, respectively. Basal and maximum OCR and were calculated according to manufacturer's instructions. Glycolysis stress test was used for evaluation of glycolysis. Cells were incubated in XF assay medium with 2 mM glutamine, followed by injections of glucose, oligomycin and 2-deoxy-glucose (2-DG) at final concentrations of 10 mM, 1 μ M and 50 mM, respectively. Basal ECAR was calculated by subtracting the acidification rate after 2-DG injection from the acidification rate after glucose injection. For IL-32 rescue cells, dead cells were removed by Optiprep (Stemcell Technologies, Vancouver, Canada) according to manufacturer's instructions and viable cells counted with the Countess Automated Cell Coulter before basal OCR and ECAR were evaluated by combining the mito stress and glycolysis stress test. The same medium supplements as for mito stress assay were used, except glucose, which was added as the first injection in the assay (final concentration of 10 mM) before injections with oligomycin, FCCP and Rotenone + antimycin+ 2-DG. Combining the injections for the two stress tests enable evaluation of both glycolysis and OXPHOS in the same cells at the same time.

#### Mitochondrial membrane potential in whole cells

For measurement of mitochondrial membrane potential IL-32 KO and WT myeloma cell lines from basal culture conditions were co-stained with 50 nM mitotracker green (Thermo Fisher) and 20 nM tetramethylrhodamine, methyl ester, perchlorate (TMRM) in serum free RPMI for 30 min at 37°C. 10 min before end of incubation FCCP was added to negative control samples (background staining) at a final concentration of 3 μM for depolarization of mitochondrial membrane. The dye was removed by two washes at 448 × g with PBS at 4°C, before resuspended in 2% FCS/PBS and analyzed by flow cytometry using LSR II (BD Biosciences) with FACS Diva software (BD Biosciences). TMRM was detected at excitation/emission 548/574 nm and mitotracker green in the 490/516 nm. The relative mitochondrial membrane potential was calculated by calculating ratio of the median fluorescence intensity (MFI) of TMRM (membrane potential) and mitotracker green (mitochondrial mass) to adjust for potential differences in mitochondrial mass between KO and WT cell lines. Further the same ratio was calculated for the FCCP (depolarized) treated sample,and was subtracted to remove the signal conferred by unspecific TMRM staining. The following formula was used:

$$\text{Nontreated}\left(\frac{MFI\;TMRM}{MFI\;\text{mitotracker green}}\right)-FCCP\left(\frac{MFI\;TMRM}{MFI\;\text{mitotracker green}}\right)\;.$$

#### Mitochondrial ROS and mitochondrial mass in whole cells

For mitochondrial ROS samples were stained mitoSOX (5 μM) for 15 min. The dye was removed by two washes at 448 × g with PBS at 4°C, before resuspended in 2% FCS/PBS and analyzed by flow cytometry. MitoSoX was detected at excitation/emission 510/580 nm using LSR II (BD Biosciences) with FACS Diva software (BD Biosciences). MFI of mitochondrial ROS was normalized with WT as reference for each separate experiment. For analysis of mitochondrial mass in whole cells, cells were stained with 50 nM mitotracker green (Thermo Fisher) in serum free RPMI for 30 min at 37°C. The dye was removed by two washes at 448 × g with PBS at 4°C, before resuspended in 2% FCS/PBS and analyzed by flow cytometry at 490/516 nm using LSR II (BD Biosciences) with FACS Diva software (BD Biosciences).

#### ATP quantification

For ATP measurements, 40,000 cells were seeded/well and ATP levels were measured by CellTiter-Glo Luminescent (CTG) Cell Viability Assay (Promega) following manufacturer's instructions. Luminescence was recorded using a Victor3 plate reader and Wallac 1420 Work Station software (PerkinElmer Inc.).

#### Isolation of mitochondria

Mitochondria from IL-32 KO and WT cells were isolated according to the protocol previously described (Lampl et al., 2015). 70–100∗10^6^ IL-32 KO and WT cells were harvested and pelleted for 8 min at 700 × g at 4°C before resuspended in PBS. Samples were then transferred to Eppendorf tubes and pelleted using a bench top centrifuge at 700 ∗g for 5 min at at 4°C. Each pellet was resuspended in 1 mL of mitochondrial isolation buffer (MIB; 200 mM sucrose, 10 mM tris/mops (PH 7.4), and 1 mM EGTA/Tris) and transferred to a small glass vessel on ice for homogenization. Cells were homogenized with a pestle for 20 passes. Homogenate was then drawn into a syringe with 26 gauge∗1/2-inch needle and expelled 5 times against the inside wall of the tube as to utilize the force for cell membrane disruption, before transferred back to the vessel and homogenized with the pestle for 20 more passes. The solution was then centrifuged for 5 min at 600 × g, 4°C in a table top centrifuge to remove cell debris and mitochondria in supernatant was pelleted with a second spin at 10,000 × g, 4°C for 5 min. Mitochondrial pellets were resuspended in MIB on ice and concentration assayed by Bradford assay followed by downstream applications.

#### Seahorse metabolic assay on isolated mitochondria

Isolated mitochondria from 70-100∗10^6^ IL-32 KO and WT cells were quantified by Bradford assay and resuspended to desired concentration in mitochondrial assay buffer (MAS) (70 mM sucrose, 220 mM mannitol, 10 mM KH_2_PO_4_, 5 mM MgCL_2_, 2 mM HEPES, 1 mM EGTA and 0.2% fatty acid free BSA) which had been supplemented with 2 mM malate, 10 mM Na-pyruvate and 5 mM glutamic acid and preadjusted to pH 7.2 at 37°C. 20 u g mitochondria were plated in each well of an XFe96 seahorse plate and the plate was spun at 2000 × g for 20 min at 4°C for attachment of the mitochondria. After centrifugation pre-varmed (37°C) MAS + substrates were added to each well to a final volume of 180 mL incubated in a non-CO_2_ incubator for 20 min, before basal oxygen consumption rate (OCR) was measured using Seahorse XF96 bioscience extracellular flux analyzer (Agilent, CA, US).

#### Mitochondrial ROS and membrane potential in isolated mitochondria

Isolated mitochondria were quantified by Bradford assay and stained for 10 min at 37°C in a 20% O_2_, 5% CO_2_ incubator with 5 mM MitoSox Red (Thermo Fisher) in MAS (70mM sucrose, 220 mM mannitol, 10 mM KH_2_PO_4_, 5 mM MgCL_2_, 2 mM HEPES, 1 mM EGTA and 0.2% fatty acid free BSA) which had been supplemented with 2 mM malate, 10 mM Na-pyruvate and 5 mM glutamic acid and preadjusted to pH 7.2 at 37°C. After staining the mitochondria were washed once in MAS and resuspended in MAS. 10 u g mitochondria/well were plated in 96-well optical plates and incubated for 60 min in 37°C (20% O_2_, 5% CO_2_ incubator). The fluorescence was assessed at with excitation 531 nm and emission 590 nm using Victor3 plate reader and Wallac 1420 Work Station software. For assessment of membrane potential, the isolated mitochondria were prepared in MAS buffer, stained with 500 nM Mitotracker Orange CMTMRos for 30 min 37°C in a 20% O_2_, 5% CO_2_ incubator before washed with MAS and seeded in 10 mg/well in 96-well optical plates and assessed immediately with excitation 531nm and emission 590 nm using Victor3 plate reader and Wallac 1420 Work Station software.

#### RNA-sequencing of IL-32 KO and WT cell lines

INA-6 IL-32 KO and WT cells were harvested from basal culture conditions and RNA was isolated using RNeasy kit (Qiagen). Samples were sequenced (18 million reads per sample) using the TruSeq Stranded mRNA library preparation kit from Illumina followed by 75bp single read sequencing on the Illumina Hiseq 4000 next machine.

#### RNA-sequencing data analyses in INA-6 KO and WT cells

The RNA-seq data were first aligned to the human genome with *STAR aligner* using the genome version GRCh38.p7 and the primary assembly (Homo_sapiens.GRCh38.DNA.primary_assembly.fa).

We used the following parameters for STAR aligner: *STAR --genomeDir GRCh38.p7/star --readFilesIn --readFilesCommand zcat --outFileNamePrefix --chimSegmentMin 30 --runThreadN 20 --outFilterMultimapNmax 20 --alignSJoverhangMin 8 --alignSJDBoverhangMin 1 --outFilterMismatchNmax 10 --outFilterMismatchNoverLmax 0.04 --alignIntronMin 20 –alignIntronMax 1000000*.

Following the alignment, the sam files were used as input into *htseq-count* to create the count table using the GFF file corresponding to the GRCh38.p7 genome assembly. For *htseq-count* we used the following parameters: *htseq-count -s no -i gene_id -t exon*.

Principle component analysis (PCA) was carried out using the *stats* package with the p*rcomp* function in R and visualized using the *ggbio* package. Differentially expressed genes were calculated using *limma-voom* in R. Scripts for PCA and limma analysis are provided in “Data and code availability”. We required genes to be expressed with at least 1 cpm in 20% of the samples. We used TMM normalization for calculating the “calcNormFactors” in limma. Differentially expressed genes between KO and WT were determined by setting a contrast in *limma-voom* such that the average of the two KO-clones were subtracted from the WT-clone: *KO1KO2_vs_WT=(0.5∗(KO1+KO2))-WT*. p values were adjusted using Benjamini-Hochberg.

The GO-analyses for the IL32- KO and WT sequencing data were performed as described below for the CoMMpass data (see figure texts for filtering of data). IL-32 isoform analysis was performed using Kallisto (v0.43.0). The Kallisto analysis were performed on the fastq-files using the following parameters: *kallisto quant -i Homo_sapiens.GRCh38.cdna.all.release-94_k31.idx -o output -b 100 -L 200 -s 20 --single -t 20 input.fastq.gz* Kallisto index was created using the following command: *kallisto index -k31 Homo_sapiens.GRCh38.cdna.all.fa.gz*.

#### RNA-sequencing data analyses in MMRF CoMMpass

RNA sequencing data (MMRF_CoMMpass_IA13a_E74GTF_Salmon_Gene_Counts) and clinical data were downloaded from the Multiple Myeloma Research Foundation CoMMpass IA13 release. RNA sequencing data from CD138^+^cells were available for 795 baseline samples from patients with MM. Data on overall survival and progression-free survival were available for all these patients. RNA-sequenced CD138^+^ cells from longitudinal samples were available for 47 samples in IA13. We analyzed IL-32 expression in 47 patients at diagnosis and first relapse time point. For survival analyses, patient samples taken at diagnosis were divided into high and low IL-32 expression based on the upper 10th percentile (n = 54; counts per million (cpm,log2)>1.52) and lower 90th percentile (n = 741, (cpm,log2)<1.52). Differentially expressed genes between high (10th percentile) and low IL32 (90th percentile) were assessed using the same percentiles, and expression-requirement of 1cpm in at least 20% of the samples. Differentially expressed genes were calculated using *limma-voom* in R using the script provided in “Data and code availability”. GO analyses were performed using R package ClusterProfiler (v3.14.3) using expressed genes as background. Cutoff for significance of genes implemented in GO was p = 0.01. Survival-analysis was performed in R, using the package “survival”. High and low IL-32 was defined as previously described and “Time” and “Status/Sensoring” were collected from the clinical data in ComMMpass. Survival curves were plotted using “ggsurvplot” in R using the package “survminer”.

All analyses were run using R version 3.6.2 (2019-12-12). Used packages with version number includes:packageVersion ("survminer")‘0.4.6’; packageVersion

("biomaRt")‘2.41.4’; packageVersion("clusterProfiler")‘3.12.0’; packageVersion("org.Hs.eg.db")‘3.8.2’; packageVersion("limma")‘3.40.6’; packageVersion("edgeR")‘3.26.8’; packageVersion("survival")‘3.1.8’; packageVersion("pheatmap")‘1.0.12’; packageVersion("ggplot2″) ‘3.2.1’.

#### Single-cell transcriptome analysis

The single cell data was download from Gene Expression Omnibus (GEO) using the accession number GSE106218. The data was analyzed as described by Ryu et al. (Ryu et al., 2020) using the *Seurat* package in *R* (Satija et al., 2015). Specifically, dimension reduction was performed using uniform manifold approximation and projection (UMAP) by using the 10 first principal components from the *FindNeighbors* and *FindClusters* functions in *Seurat*. IL-32-expressing cells were defined as cells for which at least one read for IL-32 was detected and IL-32 non-expressing cells were defined as cells for which no reads for IL32 were detected. High and low IL32 expression is defined similarly. High and low IL32 was defined within each patient for all analyses. Differentially expressed genes between IL-32-expressing cells and IL-32 non-expressing cells were detected using *the FindMarkers function in Seurat.* For each of the three patients that express IL-32, we performed differential expression analysis between cells expression high levels of IL32 (top 10% quantile) and low IL32 levels (bottom 90% quantile) to remove patient-specific biases in the analysis. The three lists of differentially expressed genes were then merged and used as input in the gene-ontology analysis. Gene ontology (GO) analysis was performed using the package clusterProfiler Yu (Yu et al., 2012) and the *enrichGO* function in *R*. The p values were adjusted using Benjamini-Hochberg method and a q-value cutoff of 0.05 was used. Genes with log2 fold change above 0.5 was used as input in the gene ontology analysis and genes expressed in at least one cells were used as background. The *simplify* function in *clusterProfiler* was used to merge similar GO-terms. The GO-terms were order by q-value and the top 20 terms were plotted using *ggplot* in R.

### Quantification and statistical analysis

Statistical analyses were performed using GraphPad Prism version 9 (GraphPad Software) unless otherwise stated. Paired or paired ratio or unpaired Student's *t* test or Wilcoxon signed-rank test were used to compare two groups. For comparison of two groups, and more than two groups with measurements over time, multiple t-tests and two-way ANOVA followed by Sidàk's or Dunnett's multiple comparisons test were used, respectively. Statistical details, including value of N (which represents independent experiments), definition of statistical significance (asterisk representing p value and cutoff values) and how data were quantified, including error bars (SD or SEM) can be found in the figure legends. p values indicated with asterisk as follows: ∗p ≤0.05, ∗∗p≤ 0.01, ∗∗∗p ≤0.001, ∗∗∗∗p ≤0.0001.

## Data Availability

• The RNA-seq data on INA-6 knock-out and wild-type cells (6 samples in total) have been uploaded to SRA with accession PRJNA769223. The metabolomics data is submitted to Mendeley Data with the title: “Metabolomics data INA-6 KO and WT” and can be viewed at Mendeley Data: https://doi.org/10.17632/dyndvz5vfn.1. • Original Western blot images have been deposited at Mendeley and are publicly available as of the date of publication at Mendeley data: https://doi.org/10.17632/hcdmjrjhft.1. • R-codes are available on Github: https://github.com/MjelleLab/IL32.git • Any additional information required to reanalyze the data reported in this paper is available from the lead contact upon request

## Supplemental information

Document S1. Figures S1–S7

Table S1. Significantly altered metabolites between two INA-6 CRISPR/Cas9 KO clones and one WT clone. Related to Figure 4

Table S2. Differentially expressed genes between two INA-6 CRISPR/Cas9 KO clones vs one WT clone. Related to Figure 4

Table S3. Differentially expressed genes between IL-32 expressing patients (upper 10th percentile) and IL-32 non-expressing patients (lower 90th percentile) in CoMMpass IA13. Related to Figure 5

Table S4. Gene ontology biological processes enriched in IL-32 expressing patients. Related to Figure 5

Table S5. Differentially expressed genes between INA-6 IL-32 CRISPR/Cas9 KO vs WT clones and between IL-32 expressing patients vs IL-32 non-expressing patients. Related to Figure 7
