# Supplementary material for: Intracellular IL-32 regulates mitochondrial metabolism, proliferation, and differentiation of malignant plasma cells
Source: iScience. 2021 Dec 13;25(1):103605. doi: 10.1016/j.isci.2021.103605 (PMC8717606; doi:10.1016/j.isci.2021.103605)
Supplement: Document S1. Figures S1–S7 [file mmc1.pdf]

iScience, Volume *25*

Supplemental information

Intracellular IL-32 regulates mitochondrial metabolism, proliferation, and differentiation of malignant plasma cells

Kristin Roseth Aass, Robin Mjelle, Martin H. Kastnes, Synne S. Tryggestad, Luca M. van den Brink, Ingrid Aass Roseth, Marita Westhrin, Muhammad Zahoor, Siv H. Moen, Tonje M. Vikene Nedal, Glenn Buene, Kristine Misund, Anne-Marit Sponaas, Qianli Ma, Anders Sundan, Richard WJ. Groen, Tobias S. Slørdahl, Anders Waage, and Therese Standal

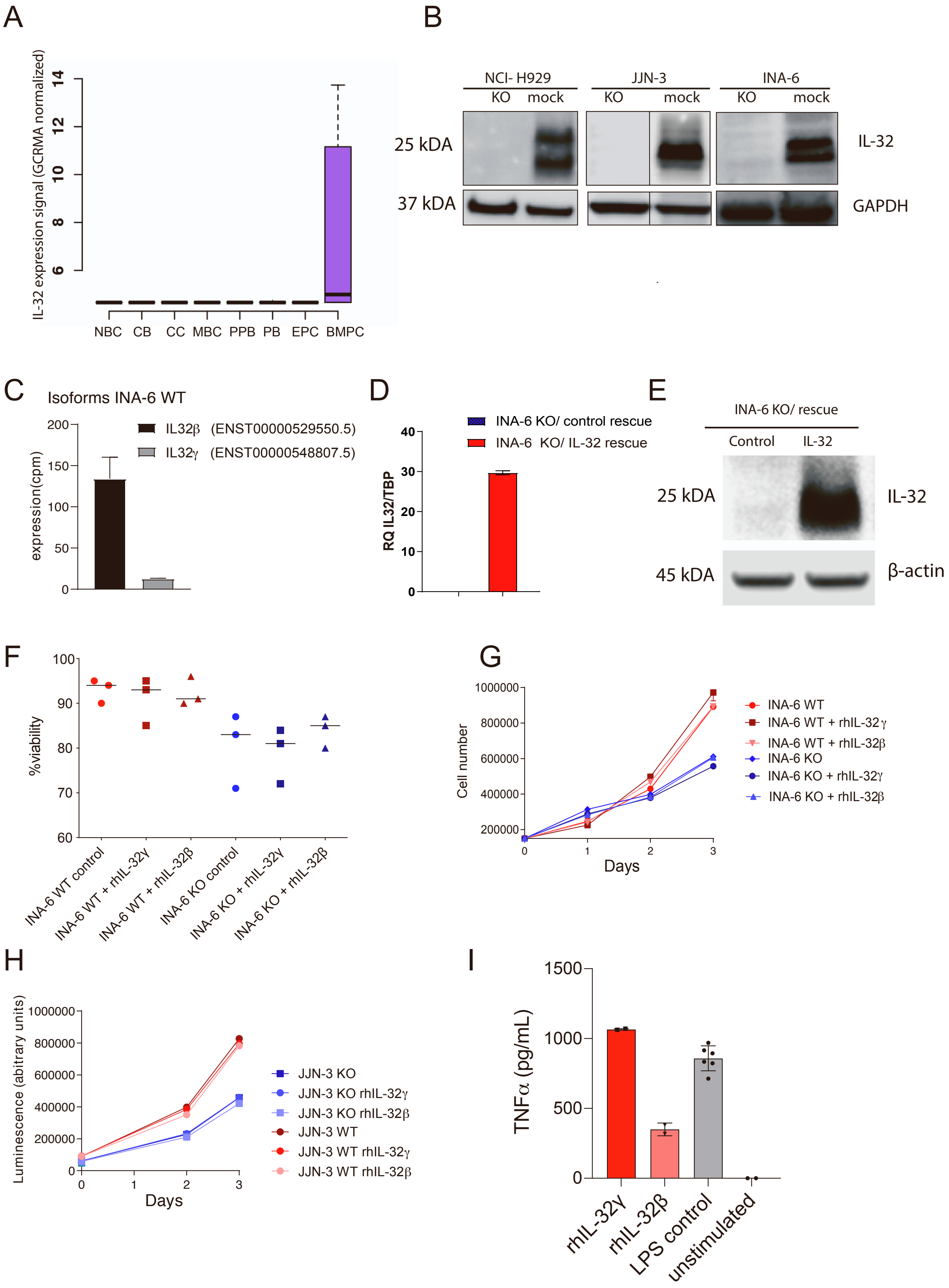

A
IL-32 expression signal (GCRMA normalized)
NBC CB CC MBC PPB PB EPC BMPC
B
NCI- H929
JJN-3
INA-6
KO mock
25 kDA
37 kDA
IL-32
GAPDH
C
Isoforms INA-6 WT
IL32β (ENST00000529550.5)
IL32γ (ENST00000548807.5)
expression(cpm)
D
INA-6 KO/ control rescue
INA-6 KO/ IL-32 rescue
RQ IL32/TBP
E
INA-6 KO/ rescue
Control
IL-32
25 kDA
45 kDA
β-actin
F
%viability
INA-6 WT control
INA-6 WT + rhIL-32γ
INA-6 WT + rhIL-32β
INA-6 KO control
INA-6 KO + rhIL-32γ
INA-6 KO + rhIL-32β
G
Cell number
Days
INA-6 WT
INA-6 WT + rhIL-32γ
INA-6 WT + rhIL-32β
INA-6 KO
INA-6 KO + rhIL-32γ
INA-6 KO + rhIL-32β
H
Luminescence (abitrary units)
Days
JJN-3 KO
JJN-3 KO rhIL-32γ
JJN-3 KO rhIL-32β
JJN-3 WT
JJN-3 WT rhIL-32γ
JJN-3 WT rhIL-32β
I
TNFα (pg/mL)
rhIL-32γ
rhIL-32β
LPS control
unstimulated

Figure S1: IL-32 expression in B-cell subtypes, IL-32 CRISPR KO/knock-in validation, isoform expression and rhIL-32 rescue stimulation experiments and biological activity. Related to Figure 1.

(A) IL-32 gene expression analyzed in Affymetrix microarray data (GCRMA normalized) of B cell subtypes derived from healthy individuals publicly available at GenomicScape.com (ID: GS-DT-2). NBC: naïve B cells (n=5), CB: centroblasts (n=4), CC: centrocytes (n=4), MBC: memory B cells (n=5), PPB: preplasmablasts (n=5), PB: plasmablasts (n=5), EPC: early plasma cells (n=5), BMPC: bone marrow plasma cells (n=5). **(B)** H929, JJN-3 and INA-6 IL-32 KO cells were generated by CRISPR/Cas9 gene editing, using lentiviral transduction (H929) and plasmid transfection (JJN3 and INA-6). Efficiency of IL32 knockout was evaluated by western blotting of IL-32. Vertical line indicates that the blot was cut to align the lanes of interest. **(C)** IL-32 isoforms in RNA-sequenced INA-6 WT mock cells analyzed by Kalisto in R. The Ensembl transcript ID is shown in brackets. **(D)** INA-6 KO/IL-32 rescue cells or rescue control cells were generated by transducing IL-32 KO cells with an IL-32 lentiviral vector or control vector, respectively. IL-32 mRNA in the cells were assessed by qPCR. Mean± SD of technical replicates of one representative of 3 experiments is shown. **(E)** Knock-in efficiency in INA-6 KO IL-32 rescue cells evaluated by western blot of IL-32. **(F)** Viability of INA-6 KO and WT mock cells treated with rhIL-32 isoforms β and γ (100 ng/mL) overnight evaluated by trypan blue staining. The mean ± SEM for 3 independent experiments is shown. Statistical significance was evaluated by two-way ANOVA and Dunnett´s multiple comparisons test for F-H. There were no significant differences. **(G)** INA-6 KO and WT mock cells were treated with rhIL-32 isoforms β and γ (100 ng/mL) and proliferation was evaluated by automated cell counting for 3 days. Each time point shows the mean ± SD of two technical replicates. **(H)** JJN-3 KO and WT mock cell proliferation with and without medium supplementation of rhIL-32 isoforms β and γ (100 ng/mL) was assessed by CTG assay. Arbitrary units were normalized to values at day 0 for each cell line. Each time point shows the mean ± SD of 3 technical replicates. **(I)** Biological activity of rhIL-32 (100 ng/mL) as evaluated by secretion of TNFα from primary macrophages. Mean ± SD of technical replicates of one experiment is shown.

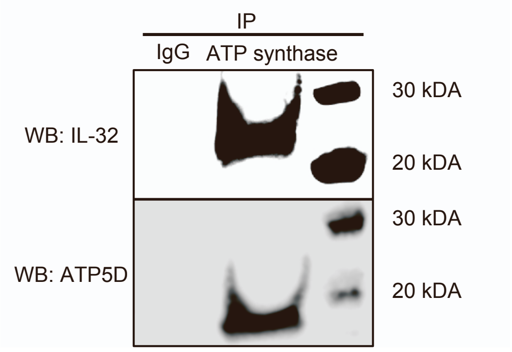

Figure S2: IL-32 detection in ATP synthase pulldown. Related to Figure 2.

CO-IP was performed by pulldown of the ATP synthase complex in INA-6 cells and IL-32 and ATP5D were detected by immunoblotting. Figure shows one representative immunoblot of 3 independent experiments.

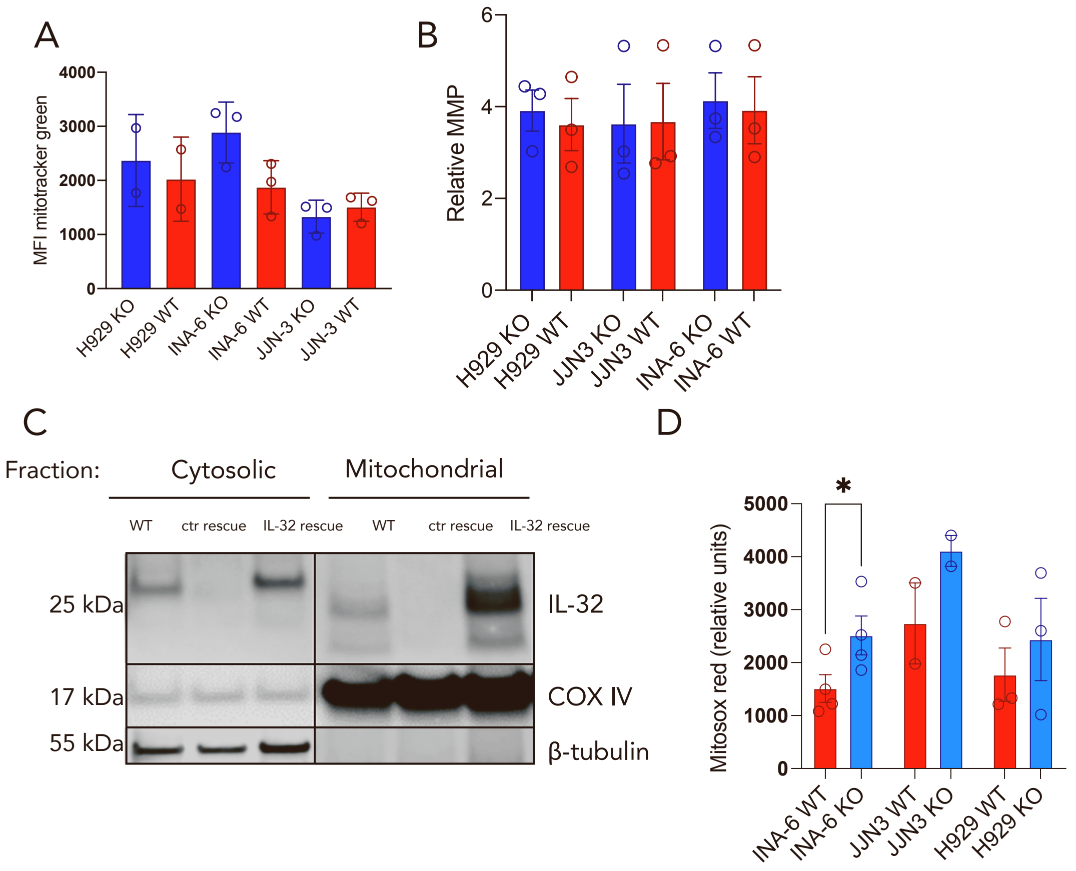

Figure S3: Mitochondrial mass, mitochondrial membrane potential and mitochondrial ROS in IL-32 KO cell lines and isolated mitochondria. Detection of IL-32 in the mitochondrial fraction of INA-6 IL-32 KO/rescue cells. Related to Figure 3.

(A) Mitochondrial mass in IL-32 KO and WT cell lines quantified by mitotracker green. Data shown are mean ± SEM of 3 independent experiments. The differences were not significant. **(B)** Relative mitochondrial membrane potential (MMP) in myeloma IL-32 KO and WT cell lines quantified by TMRM/ mitotracker green flow cytometry. The results are presented as mean ± SEM of 3 independent experiments. There were no significant differences. **(C)** Immunoblot of IL-32 in mitochondrial and cytosolic fraction of INA-6 KO/ IL-32 rescue cells and INA-6 KO/rescue control cells. **(D)** Mitochondrial ROS in isolated mitochondria from myeloma KO and WT cell lines quantified by MitoSox Red. The results are presented as the mean ± SEM of biological replicates. P-values in A, B and D were analyzed by unpaired Student´s t-test. * $P \leq 0.05$

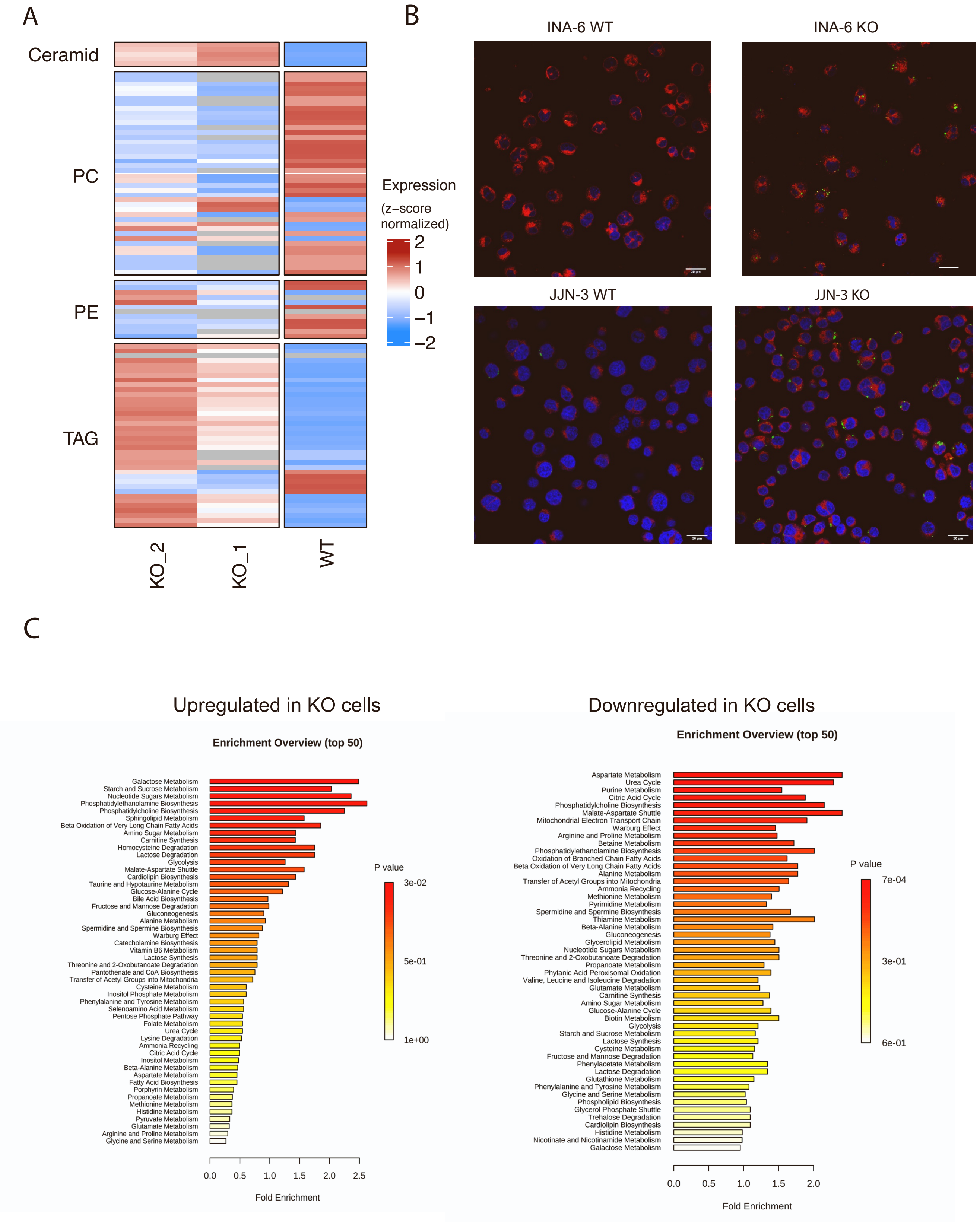
A
Ceramid
PC
PE
TAG
KO_2
KO_1
WT
Expression
(z–score
normalized)
2
1
0
–1
–2
B
INA-6 WT
INA-6 KO
JJN-3 WT
JJN-3 KO
C
Upregulated in KO cells
Enrichment Overview (top 50)
Galactose Metabolism
Starch and Sucrose Metabolism
Nucleotide Sugars Metabolism
Phosphatidylethanolamine Biosynthesis
Phosphatidylcholine Biosynthesis
Sphingolipid Metabolism
Beta Oxidation of Very Long Chain Fatty Acids
Amino Sugar Metabolism
Carnitine Synthesis
Homocysteine Degradation
Lactose Degradation
Glycolysis
Malate-Aspartate Shuttle
Cardiolipin Biosynthesis
Taurine and Hypotaurine Metabolism
Glucose-Alanine Cycle
Bile Acid Biosynthesis
Fructose and Mannose Degradation
Gluconeogenesis
Alanine Metabolism
Spermidine and Spermine Biosynthesis
Warburg Effect
Catecholamine Biosynthesis
Vitamin B6 Metabolism
Lactose Synthesis
Threonine and 2-Oxobutanoate Degradation
Pantothenate and CoA Biosynthesis
Transfer of Acetyl Groups into Mitochondria
Cysteine Metabolism
Inositol Phosphate Metabolism
Phenylalanine and Tyrosine Metabolism
Selenoamino Acid Metabolism
Pentose Phosphate Pathway
Folate Metabolism
Urea Cycle
Lysine Degradation
Ammonia Recycling
Citric Acid Cycle
Inositol Metabolism
Beta-Alanine Metabolism
Aspartate Metabolism
Fatty Acid Biosynthesis
Porphyrin Metabolism
Propanoate Metabolism
Methionine Metabolism
Histidine Metabolism
Pyruvate Metabolism
Glutamate Metabolism
Arginine and Proline Metabolism
Glycine and Serine Metabolism
0.0
0.5
1.0
1.5
2.0
2.5
Fold Enrichment
P value
3e-02
5e-01
1e+00
Downregulated in KO cells
Enrichment Overview (top 50)
Aspartate Metabolism
Urea Cycle
Purine Metabolism
Citric Acid Cycle
Phosphatidylcholine Biosynthesis
Malate-Aspartate Shuttle
Mitochondrial Electron Transport Chain
Warburg Effect
Arginine and Proline Metabolism
Betaine Metabolism
Phosphatidylethanolamine Biosynthesis
Oxidation of Branched Chain Fatty Acids
Beta Oxidation of Very Long Chain Fatty Acids
Alanine Metabolism
Transfer of Acetyl Groups into Mitochondria
Ammonia Recycling
Methionine Metabolism
Pyrimidine Metabolism
Spermidine and Spermine Biosynthesis
Thiamine Metabolism
Beta-Alanine Metabolism
Gluconeogenesis
Glycerolipid Metabolism
Nucleotide Sugars Metabolism
Threonine and 2-Oxobutanoate Degradation
Propanoate Metabolism
Phytanic Acid Peroxisomal Oxidation
Valine, Leucine and Isoleucine Degradation
Glutamate Metabolism
Carnitine Synthesis
Amino Sugar Metabolism
Glucose-Alanine Cycle
Biotin Metabolism
Glycolysis
Starch and Sucrose Metabolism
Lactose Synthesis
Cysteine Metabolism
Fructose and Mannose Degradation
Phenylacetate Metabolism
Lactose Degradation
Glutathione Metabolism
Phenylalanine and Tyrosine Metabolism
Glycine and Serine Metabolism
Phospholipid Biosynthesis
Glycerol Phosphate Shuttle
Trehalose Degradation
Cardiolipin Biosynthesis
Histidine Metabolism
Nicotinate and Nicotinamide Metabolism
Galactose Metabolism
0.0
0.5
1.0
1.5
2.0
Fold Enrichment
P value
7e-04
3e-01
6e-01

Figure S4. Heatmap of differentially expressed lipid species between INA-6 KO and WT cells. Lipid droplet staining of IL-32 KO cells and pathway enrichment analysis of differentially expressed metabolites between KO and WT cells. Related to Figure 4.

(A) Heatmap showing significantly altered ceramides, phosphatidylcholine (PC), phosphatidylethanolamine (PE) and triglyceride (TAG) species in two INA-6 KO clones (KO1, KO2) and WT mock cells. The details on the lipid species within each class can be found in Table S1. **(B)** Lipid droplets in INA-6 and JJN-3 KO and WT mock cells, stained with Nile Red. Polar lipids (red) were excited at 590 nm (600–700 nm) and neutral lipids (green) at 488 nm (500–580 nm). Hoechst stain of nucleus in blue. Confocal imaging was performed with a Leica TCS SP8 STED 3X using a 63X 1.4 (oil) objective and LAS X software. Scale bar: 20 μM. **(C)** Pathway enrichment of significantly downregulated and upregulated metabolites (See Table S1) in INA-6 KO cells compared to WT cells using Metaboanalyst 4.0.

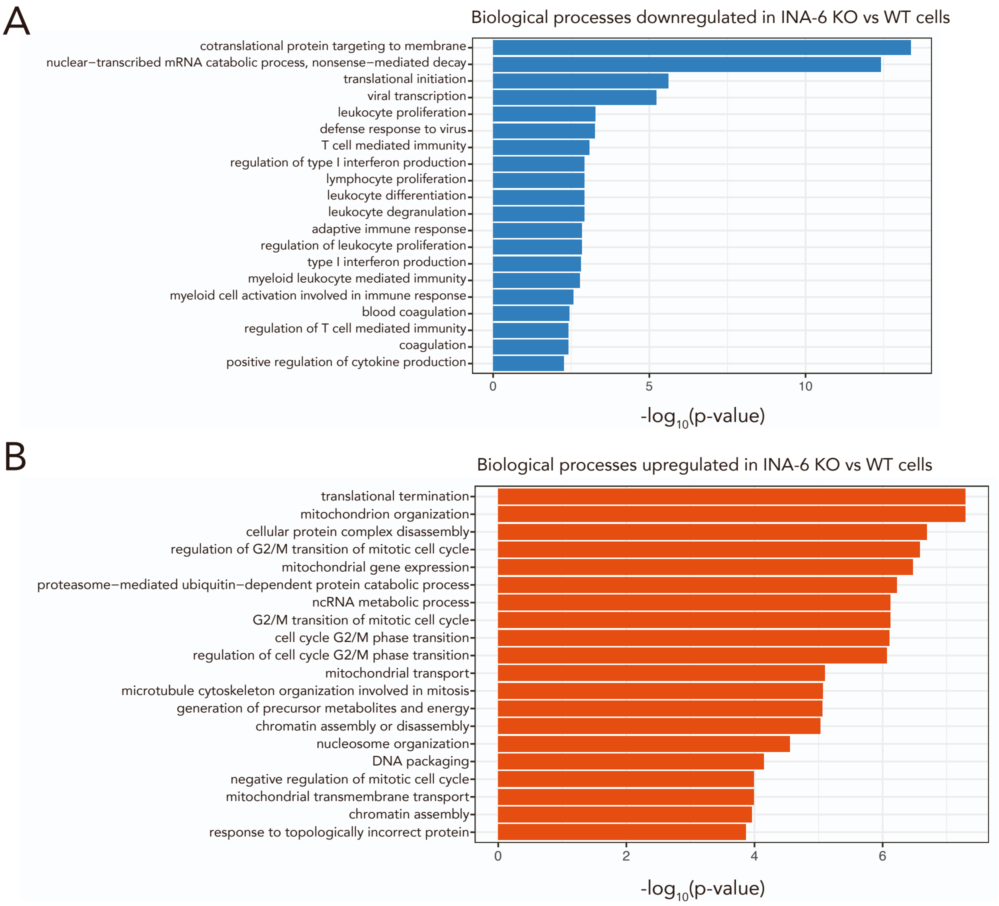

Figure S5: Gene ontology (GO) enrichment analysis of differentially expressed genes between INA-6 IL-32 KO and WT cells. Related to Figure 4.

GO-analysis of the differentially expressed genes (Benjamini-Hochberg-adjusted P-value < 0.05; log2 fold change >0 and <0 for up- and -down-regulated genes, respectively) between INA-6 KO and WT mock cell lines. The figures show the top 20 significantly enriched biological processes downregulated **(A)** or upregulated **(B)** in KOs vs WT mock. The GO terms are ordered by the Benjamini-Hochberg adjusted P-values.

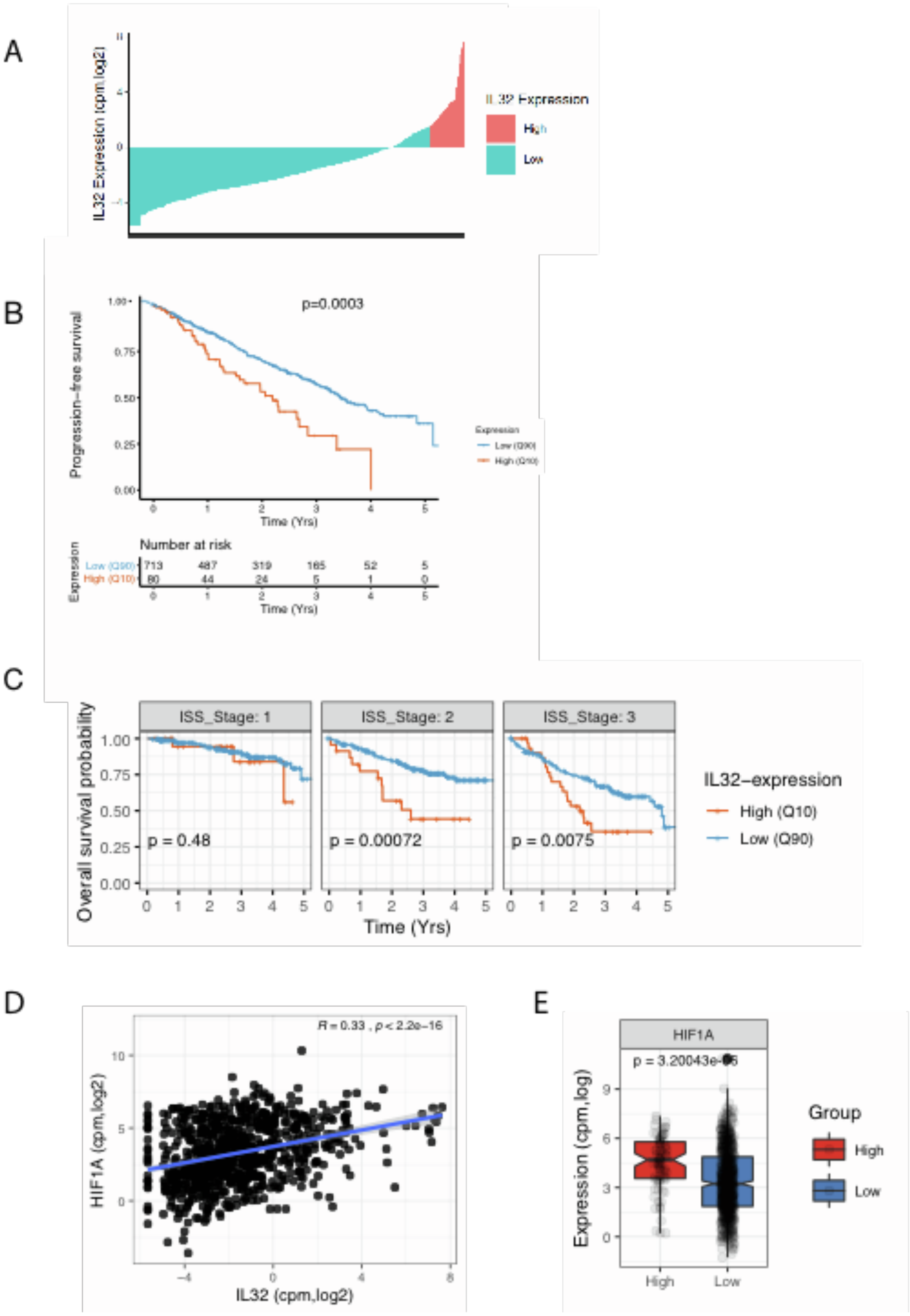
A
B
C
D
E
IL32 Expression (cpm,log2)
IL32 Expression
High
Low
p=0.0003
Progression-free survival
Time (Yrs)
Expression
Low (Q90)
High (Q10)
Number at risk
713 487 319 165 52 5
80 44 24 5 1 0
ISS_Stage: 1
ISS_Stage: 2
ISS_Stage: 3
Overall survival probability
p = 0.48
p = 0.00072
p = 0.0075
IL32-expression
High (Q10)
Low (Q90)
Time (Yrs)
R = 0.33 , p < 2.2e-16
HIF1A (cpm,log2)
IL32 (cpm,log2)
HIF1A
Expression (cpm,log)
Group
High
Low

Figure S6: IL-32 is an independent prognostic factor and associated with HIF1A in patients. Related to Figure 5.

(A) IL-32 expression in all patients from CoMMpass IA13 (n= 792), upper 10th percentile (n= 80, log2 cpm> 1.52) colored in pink. **(B)** Progression- free survival of IL-32 expressing patients (10th percentile) compared to nonexpressing patients (90th percentile) in the IA13 CoMMpass dataset P= 0.0003, using Cox proportional-hazards regression model. **(C)** Assessment of IL-32 as an independent prognostic factor for overall survival in IA13. IL-32 expressing patients (10th percentile) compared to non-expressing patients (90th percentile) at different ISS stages (stage I, stage II, or stage III disease). Adjustment for ISS stage was performed using multivariate Cox-regression, using the coxph function in R. **(D)** Correlation between IL-32 and HIF1A in CoMMpass IA13, evaluated by Pearson correlation coefficient.**(E)** HIF1A gene expression in IL-32-expressing patients (upper 10th percentile) compared to non-expressing patients (lower 90th percentile) in CoMMpass IA13. Significance was determined by limma in R.

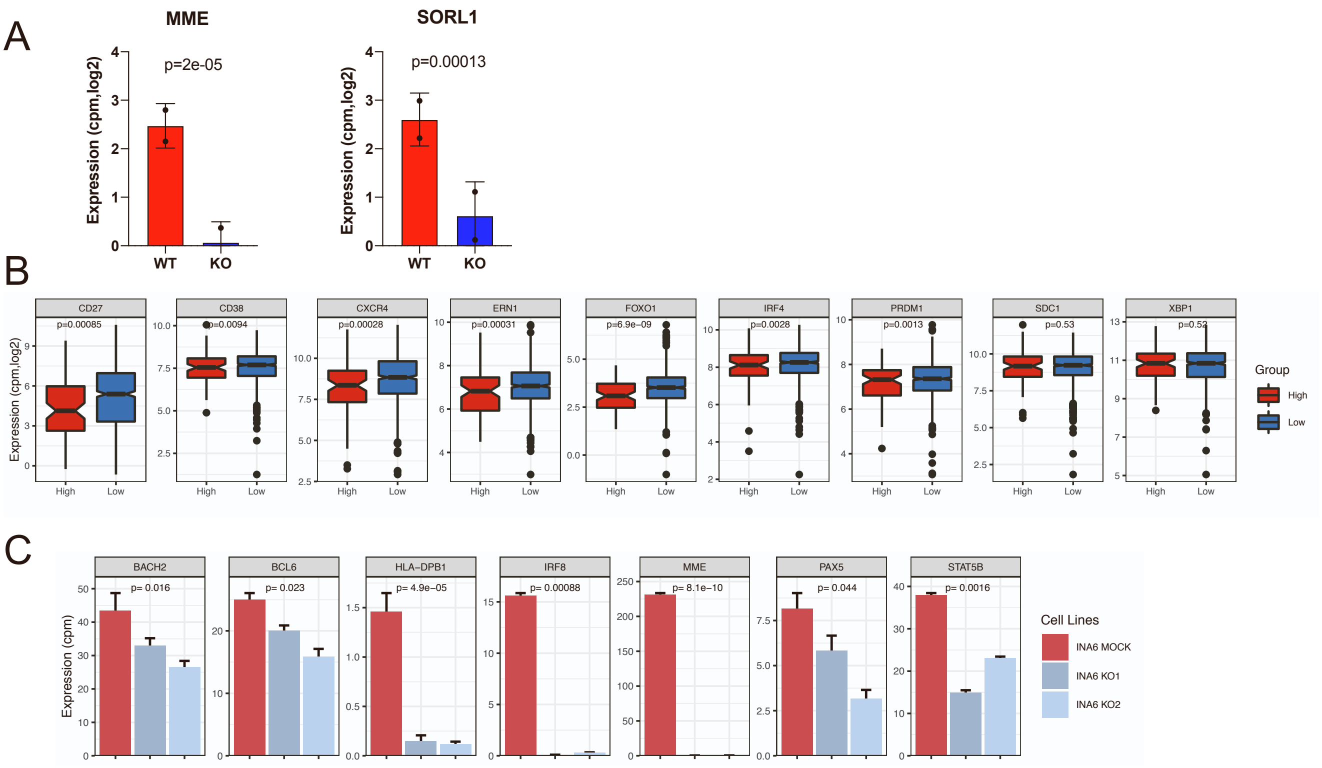

Figure S7: IL-32 expression is associated with an immature phenotype. Related to Figure 7.

(A) Gene expression of MME and SORL1 in H929 IL-32 KO and WT mock cells. Significance calculated by limma t-test with Benjamini-Hochberg-adjusted p-values in R.

(B) Evaluation of gene expression of typical mature plasma cell markers (based on a literature search) in IL-32 expressing patients (upper10th percentile) compared to non-expressing patients (lower 90th percentile) in CoMMpass IA13. Significance analyzed by limma in R.

(C) Genes associated with less differentiated stages of B-cell maturation downregulated in INA-6 KO cells assessed from RNA-sequencing data of INA-6 KO1, KO2 and WT mock cells. P-values analyzed by limma in R.
